# Comparative kinase and cancer cell panel profiling of kinase inhibitors approved for clinical use from 2018 to 2020

**DOI:** 10.3389/fonc.2022.953013

**Published:** 2022-09-14

**Authors:** Jeffrey J. Kooijman, Wilhelmina E. van Riel, Jelle Dylus, Martine B. W. Prinsen, Yvonne Grobben, Tessa J. J. de Bitter, Antoon M. van Doornmalen, Janneke J. T. M. Melis, Joost C. M. Uitdehaag, Yugo Narumi, Yusuke Kawase, Jeroen A. D. M. de Roos, Nicole Willemsen-Seegers, Guido J. R. Zaman

**Affiliations:** ^1^ Oncolines B.V., Oss, Netherlands; ^2^ Netherlands Translational Research Center B.V., Oss, Netherlands; ^3^ Carna Biosciences, Inc., Kobe, Japan

**Keywords:** cancer cell line, biochemical assay, indication expansion, cell viability assay, kinase inhibitor, drug profiling

## Abstract

During the last two decades, kinase inhibitors have become the major drug class for targeted cancer therapy. Although the number of approved kinase inhibitors increases rapidly, comprehensive *in vitro* profiling and comparison of inhibitor activities is often lacking in the public domain. Here we report the extensive profiling and comparison of 21 kinase inhibitors approved by the FDA for oncology indications since June 2018 and 13 previously approved comparators on panels of 255 biochemical kinase assays and 134 cancer cell line viability assays. Comparison of the cellular inhibition profiles of the EGFR inhibitors gefitinib, dacomitinib, and osimertinib identified the uncommon *EGFR* p.G719S mutation as a common response marker for EGFR inhibitors. Additionally, the FGFR inhibitors erdafitinib, infigratinib, and pemigatinib potently inhibited the viability of cell lines which harbored oncogenic alterations in *FGFR1-3*, irrespective of the specific clinical indications of the FGFR inhibitors. These results underscore the utility of *in vitro* kinase inhibitor profiling in cells for identifying new potential stratification markers for patient selection. Furthermore, comparison of the *in vitro* inhibition profiles of the RET inhibitors pralsetinib and selpercatinib revealed they had very similar biochemical and cellular selectivity. As an exception, an *NTRK3* fusion-positive cell line was potently inhibited by pralsetinib but not by selpercatinib, which could be explained by the targeting of TRK kinases in biochemical assays by pralsetinib but not selpercatinib. This illustrates that unexpected differences in cellular activities between inhibitors that act through the same primary target can be explained by subtle differences in biochemical targeting. Lastly, *FLT3*-mutant cell lines were responsive to both FLT3 inhibitors gilteritinib and midostaurin, and the PI3K inhibitor duvelisib. Biochemical profiling revealed that the FLT3 and PI3K inhibitors targeted distinct kinases, indicating that unique dependencies can be identified by combined biochemical and cellular profiling of kinase inhibitors. This study provides the first large scale kinase assay or cell panel profiling study for newly approved kinase inhibitors, and shows that comprehensive *in vitro* profiling of kinase inhibitors can provide rationales for therapy selection and indication expansion of approved kinase inhibitors.

## Introduction

Kinases are the major anticancer drug target class of the 21^st^ century (1) with nearly 60 small molecule kinase inhibitors approved for clinical use in the first two decades (2, 3). While there are more than 500 kinases encoded by the human genome (4), currently approved kinase inhibitors for cancer treatment act primarily through approximately 20 different targets. Key to the success of kinase inhibitor therapy has been the simultaneous development of selective inhibitors and biomarker assays to enable the selection of patients that are most likely to respond to these drugs in the clinic. Well-known examples of clinically approved biomarkers are the Philadelphia chromosome for treatment of leukemias with imatinib (5), activating mutations in the epidermal growth factor receptor (*EGFR*) gene for treatment of non-small cell lung cancer (NSCLC) with gefitinib (6), activating mutations in the B-Raf proto-oncogene (*BRAF*) for treatment of metastatic melanoma with vemurafenib (7), and gene fusions resulting in constitutive activity of the anaplastic lymphoma kinase (ALK) for treatment of NSCLC with crizotinib (8). Biomarkers that correlate with intrinsic resistance of tumors to targeted therapies have also been identified. For instance, mutations in the Kirsten rat sarcoma virus (*KRAS*) oncogene predict resistance against anti-EGFR therapy in colorectal cancer and are used to exclude patients from treatment with these agents (9–11).

The ability of kinase inhibitors to inhibit tumor cell growth can be measured *in vitro* in cell viability assays. Novel predictive drug response biomarkers can be identified by profiling compounds on a large cell line panel and subsequently relating drug sensitivity to genomic information of the cell lines (12–16). In this way, we previously identified novel genomic and transcriptomic biomarkers for several approved kinase inhibitors (15, 17, 18). Additionally, head-to-head comparison of the selectivity and potency of inhibitors in a broad panel of cancer cell line viability assays can reveal similarities or differences in their biochemical mechanism of action (19, 20).

In two earlier studies, we compared the kinase selectivity and the cell panel profiles of all kinase inhibitors approved by the Food and Drug Administration (FDA) until May 2018 (15, 17). Here we present a head-to-head comparison of the biochemical and cellular selectivity and potency profiles of 21 newly FDA approved kinase inhibitors since June 2018 and 13 previously approved kinase inhibitors acting on the same targets. Eleven of the 21 newly approved inhibitors have not been profiled in previous large-scale kinase profiling studies (21, 22), while seven inhibitors were not included in earlier large scale cancer cell line panel profiling datasets, such as the Genomics of Drug Sensitivity in Cancer, Cancer Therapeutics Response Portal, and PRISM datasets (23–26). Our cell panel profiling data confirm FDA-approved stratification markers and reveal potential novel predictive biomarkers, for instance for the EGFR and fibroblast growth factor receptor (FGFR) inhibitors. Additionally, by combining cellular and biochemical profiling data, relevant activities and selectivities of several kinase inhibitors were discovered that may be explored to expand the application of kinase inhibitors for other therapeutic indications, such as the tropomyosin receptor kinase (TRK) inhibitor entrectinib for the treatment of FMS-like tyrosine kinase 3 (FLT3)-mutant acute myeloid leukemia (AML).

## Materials and methods

### Inhibitors

All kinase inhibitors were purchased from commercial vendors (as summarized in **Table S1** **)** and stored as solids at 4 °C. Before experiments, compounds were dissolved in 100% dimethyl sulfoxide (DMSO).

### Kinase assays

Compounds were profiled on a panel of 255 wild-type kinases in either mobility shift assays (MSA) or immobilized metal ion affinity particle (IMAP) assays at a compound concentration of 1 µmol/L and an ATP concentration within 2-fold of the affinity for ATP (*K*_M,ATP_) of every individual kinase (27). Half-maximal inhibitory concentrations (IC_50_) on primary and secondary kinase targets were determined in duplicate 10-point dilution series of compounds in MSA for most kinases. For MEK1 and MEK2, inhibition of enzymatic activity was measured in enzyme-linked immunosorbent assays (ELISA).

### Kinome tree biochemical selectivity

Percentage inhibition values at 1 µmol/L inhibitor were grouped into four categories for the 76 tyrosine kinases included in the panel of 255 kinases (group 1: > 95%, group 2: ≥ 90% & < 95%, group 3: ≥ 50% & < 90%, and group 4: ≤ 50% inhibition). Kinome trees were generated using Coral (28).

### Cancer cell lines

Cell lines were purchased from the American Type Culture Collection (ATCC) (Manassas, VA, USA), German Collection of Microorganisms and Cell Cultures (DSMZ) (Braunschweig, Germany), Japanese Collection of Research Bioresources (JCRB) (Ibaraki city, Osaka, Japan), or RIKEN BioResource Research Center (Tsukuba, Ibaraki, Japan), as indicated in **Table S2**. Cell lines were propagated in the base cell culture medium as indicated in **Table S2**. Cell line-specific essential supplements were added to the culture media. All experiments were carried out within ten passages of the original vials. Authenticity of the cell lines was confirmed by short tandem repeat analysis at the respective provider.

### Cell viability assays

The effect of compounds on cell viability was determined by measuring intracellular ATP content as an indirect readout of cell number (15). Cells were seeded in a 384-well plate at an optimized density to ensure unrestricted growth and maximum signal at the end of the viability assay. After 24 hours incubation, the starting cell number was determined by adding ATPlite 1Step (PerkinElmer, Groningen, the Netherlands) to each well and recording luminescence on an Envision multimode reader (PerkinElmer, Waltham, MA). Kinase inhibitor stock solutions in DMSO (as indicated in **Table S1**) were diluted in √10-fold steps in 100% DMSO to obtain a 9-point dilution series and were further diluted 31.6-fold in 20 mM HEPES buffer, before directly adding the dilution series in duplicate to the incubated culture plates with cells, which further diluted the compound solution 10-fold. A vehicle-treated control (0.4% (v/v) DMSO) was included in quadruplicate on the same assay plate to determine maximum cell growth. The final DMSO concentration was 0.4% (v/v) in all wells. After incubation for another 72 hours, ATP content in each well was measured as described previously. Cell line doublings were determined by relating the vehicle-treated control to the starting cell number. When the cell doubling deviated > 2-fold from the historic doubling as determined in multiple independent experiments, the experiment was invalidated and repeated. Percentage cell viability at each inhibitor concentration was determined by relating the inhibitor-treated conditions to the vehicle-treated control. IC_50_ values of kinase inhibitors were calculated by fitting a 4-parameter logistic curve to the percentage viability values using IDBS XLfit5 (IDBS, Guildford, United Kingdom). All curves were visually inspected and submitted to an F-test as implemented in XLfit5. Curves with F > 1.5 were invalidated. For some compounds, biphasic curves were measured in one or more cell lines, indicating a dual mechanism of action in these cell lines (29). In these cases, the most potent effect was fitted. In case the concentrations of the initial dilution range of an inhibitor were too high in a certain cell line, a new dilution series was generated using a diluted stock solution and retested on the cell line, as described earlier. For all bioinformatic analyses, ^10^log(IC_50_ [nmol/L]) values were used. The IC_50_ was limited to the maximum tested inhibitor concentration when the IC_50_ exceeded the maximum tested concentration. Reported average IC_50_ values indicate the geometric average.

### Clustering of cell panel viability data

The ^10^logIC_50_ values of the kinase inhibitors on 134 cancer cell lines were compared by hierarchical clustering with the Ward method, using 1 – Pearson correlation (*r*) as clustering distance, as described before (19).

### Annotation of genomic alterations in cell lines

Cell lines were classified as having an alteration in a specific cancer gene if at least one allele was altered by point mutation, insertion, deletion, fusion, or amplification. The mutation, fusion, and amplification status of the cell lines was retrieved from the COSMIC cell lines project (version 80) (23), Cancer Cell Line Encyclopedia (CCLE), DepMap (version 22Q1) (16), and Cellosaurus (30) databases. In order to ensure that genomic alterations included in subsequent analyses were relevant to cancer growth and drug response, mutations were only included when they were either reported as a hotspot mutation in Cancer Hotspots (31), described as oncogenic and gain-of-function in OncoKB or JAX CKB, or described as pathogenic in the literature. Although the bladder carcinoma cell line J82 harbors an activating *FGFR3* p.K650E mutation, it does not express the FGFR3 protein on immunoblot and does not respond to FGFR inhibition (32). J82 was therefore annotated as *FGFR3* wild-type.

### Gene expression-based predictive biomarker analysis

Gene expression profiles of 19,177 genes were retrieved from the DepMap database (version 22Q1) (16). Cell lines which had ‘Engineered’ listed as primary disease indication were removed, resulting in a final number of 1379 cell lines for which gene expression data were available. For each of the 19,177 genes, Z-scores were calculated over all cell lines. This allowed for identification of genes which were highly expressed in a certain cell line, compared to all other cell lines.

### Drug combination assays

Cell viability assays were performed as described above. Kinase inhibitors were diluted in 6-point dilution series. The 6-point concentration ranges were selected to optimally capture the full dose-response range of each inhibitor in the cell lines of interest, based on the 9-point dilution series of the single agents. The inhibitors were mixed in a 6x6 combination matrix design, and the 6-point dilution series of the single inhibitors were included as reference. Each concentration of both the combination matrix and single compounds were profiled in quadruplicate wells. Synergy scores were calculated by the Zero Interaction Potency (ZIP) method, using the *synergyfinder* R package (version 3.2.10) (33). The ZIP score indicates the percentage of additional cell line response induced by the combination compared to the expected response based on the two single agents (33). A ZIP score > 10 was considered synergistic, from -10 to 10 was considered additive, while < -10 was considered antagonistic.

### Statistical analyses

All statistical analyses were performed in R (version 4.1.2), unless otherwise indicated. A two-sided Mann-Whitney test was performed for two-group comparisons. A *p*-value < 0.05 was considered significant. Multivariate analysis of variance (MANOVA) was performed for multi-group comparison. Benjamini-Hochberg corrected *p*-values < 0.2 were considered significant.

## Results and discussion

### Targets of recently approved kinase inhibitors

From June 2018 to February 2021, a total of 20 small molecule kinase inhibitors were approved for clinical use in cancer (**Table 1**). Several of these inhibitors target previously addressed kinases, such as ALK (lorlatinib) (34) and EGFR (dacomitinib) (35). Various other kinases have been successfully addressed for the first time with small molecule inhibitors, such as colony stimulating factor 1 receptor (CSF1R) (pexidartinib) (36), the hepatocyte growth factor receptor kinase MET (capmatinib) (37) and FGFR2-3 (erdafitinib and pemigatinib) (38, 39). The FGFR inhibitor infigratinib was only very recently approved (40, 41). Although this study is focused on kinase inhibitors approved by the FDA between 2018 and 2020, we decided to also include infigratinib in the current study, considering the extensive development history of this inhibitor. The selective TRK inhibitor larotrectinib was the first kinase inhibitor approved to target a specific genomic alteration regardless of tumor origin (42), a concept known as a tissue-agnostic indication. A second TRK inhibitor, entrectinib, was later approved for the same tissue-agnostic indication of neurotrophic tyrosine receptor kinase (*NTRK*) fusion-positive solid tumors. The progress towards tissue-agnostic indications underscores the importance of identifying suitable biomarkers for successfully advancing the development of kinase inhibitors.

**Table 1:** Overview of kinase inhibitors profiled in the current study.

Generic name	Trade name	Clinical indication	First approval
gefitinib	Iressa	*EGFR* p.L858R or exon 19 mutant NSCLC	May 2003
dacomitinib	Vizimpro	*EGFR* p.L858R or exon 19 mutant NSCLC	September 2018
osimertinib	Tagrisso	*EGFR* p.L858R, exon 19 or p.T790M mutant NSCLC	November 2015
erdafitinib	Balversa	*FGFR2*- or *FGFR3*-altered urothelial carcinoma	April 2019
infigratinib	Truseltiq	*FGFR2* fusion-positive cholangiocarcinoma	May 2021
pemigatinib	Pemazyre	*FGFR2* fusion-positive cholangiocarcinoma	April 2020
crizotinib	Xalkori	ALK- or ROS1-positive NSCLC and ALK-positive ALCL	August 2011
ceritinib	Zykadia	ALK-positive NSCLC	April 2014
alectinib	Alecensa	ALK-positive NSCLC	December 2015
brigatinib	Alunbrig	ALK-positive NSCLC	April 2017
lorlatinib	Lorbrena	ALK-positive NSCLC	November 2018
capmatinib	Tabrecta	*MET* exon 14 skipping mutant NSCLC	May 2020
entrectinib	Rozlytrek	*NTRK* fusion-positive solid tumors and ROS1-positive NSCLC	August 2019
larotrectinib	Vitrakvi	*NTRK* fusion-positive solid tumors	November 2018
pralsetinib	Gavreto	*RET* fusion-positive NSCLC and *RET* fusion-positive or mutant thyroid cancer	September 2020
selpercatinib	Retevmo	*RET* fusion-positive NSCLC and *RET* fusion-positive or mutant thyroid cancer	May 2020
midostaurin	Rydapt	*FLT3-*mutant AML, ASM, SM-AHN, and MCL	April 2017
gilteritinib	Xospata	*FLT3-*mutant AML	November 2018
pexidartinib	Turalio	Tenosynovial giant cell tumor	August 2019
duvelisib	Copiktra	Various hematological indications	September 2018
ibrutinib	Imbruvica	Various hematological indications	November 2013
acalabrutinib	Calquence	Various hematological indications	October 2017
zanubrutinib	Brukinsa	Mantle cell lymphoma	November 2019
tucatinib	Tukysa	HER2-positive breast cancer	April 2020
avapritinib	Ayvakit	*PDGFRA* p.D842V mutant GIST and advanced systemic mastocytosis	January 2020
ripretinib	Qinlock	Advanced GIST	May 2020
dabrafenib	Tafinlar	*BRAF* p.V600E/K mutant melanoma and *BRAF* p.V600E mutant NSCLC	May 2013
encorafenib	Braftovi	*BRAF* p.V600E/K mutant melanoma and *BRAF* p.V600E mutant CRC	June 2018
vemurafenib	Zelboraf	*BRAF* p.V600E mutant melanoma	August 2011
binimetinib	Mektovi	*BRAF* p.V600E/K mutant melanoma	June 2018
cobimetinib	Cotellic	*BRAF* p.V600E/K mutant melanoma	November 2015
selumetinib	Koselugo	Neurofibromatosis type 1	April 2020
trametinib	Mekinist	*BRAF* p.V600E/K mutant melanoma and *BRAF* p.V600E mutant NSCLC	May 2013
alpelisib	Piqray	*PIK3CA*-mutant, HR-positive, HER2-negative breast cancer	May 2019

NSCLC, non-small cell lung cancer; ALCL, anaplastic large cell lymphoma; AML, acute myeloid leukemia; ASM, aggressive systemic mastocytosis; SM-AHN, systemic mastocytosis with associated hematological neoplasm; MCL, mast cell leukemia; GIST, gastrointestinal stromal tumor; CRC, colorectal cancer.

Inhibitors are ordered as discussed in this work.

### Kinase and cell panel profiling

In this study, the 21 newly-approved inhibitors and 13 previously approved comparators (**Table 1**) were profiled on a biochemical assay panel of 255 wild-type kinases at a single concentration of 1 µmol/L in order to compare the selectivity between inhibitors (**Table S3**). Additionally, the IC_50_ values of the compounds on their primary target and relevant secondary kinase targets were determined in 10-point dose-response curves which allows for comparing the potency of similar inhibitors on their primary or secondary targets (**Table 2**).

**Table 2:** Biochemical potencies (IC_50_ in nmol/L) of recently approved kinase inhibitor drugs (2018 - 2020) and previously approved comparators.

Generic name	IC_50_ values on primary and secondary target(s)									
gefitinib	**EGFR**	0.41	**HER2**	49	**HER4**	4.7				
dacomitinib	**EGFR**	0.27	**HER2**	6.0	**HER4**	0.6				
osimertinib	**EGFR^1^**	25	**HER2**	18	**HER4**	3.0				
erdafitinib	**FGFR1**	0.49	**FGFR2**	0.46	**FGFR3**	0.34	**FGFR4**	2.0		
pemigatinib	**FGFR1**	0.62	**FGFR2**	0.42	**FGFR3**	0.92	**FGFR4**	8.2		
infigratinib^2^	**FGFR1**	0.54	**FGFR2**	0.45	**FGFR3**	0.61	**FGFR4**	24		
crizotinib	**ALK^1^**	1.9	**ROS^3^**	2.3	**MET^1^**	4.6				
ceritinib^1^	**ALK**	0.64								
alectinib^1^	**ALK**	0.88								
brigatinib^1^	**ALK**	0.62								
lorlatinib	**ALK**	0.54	**ROS**	0.16						
capmatinib	**MET**	2.1	**ALK**	>1000						
entrectinib	**TRKA**	0.52	**TRKB**	0.67	**TRKC**	0.71	**ROS**	1.0	**ALK**	2.6
larotrectinib	**TRKA**	0.91	**TRKB**	1.4	**TRKC**	1.4	**ROS**	135	**ALK**	>1000
pralsetinib	**RET**	0.83	**FGFR1**	11	**FGFR2**	27	**FGFR3**	50	**FGFR4**	183
selpercatinib	**RET**	0.45	**FGFR1**	46	**FGFR2**	16	**FGFR3**	50	**FGFR4**	260
midostaurin^1^	**FLT3**	3.3								
gilteritinib	**FLT3**	0.40	**ALK**	0.74						
pexidartinib	**FMS**	125	**KIT**	132	**FLT3**	737				
duvelisib^4^	**PI3Kδ**	0.023	**PI3Kγ**	0.44	**PI3Kα**	32				
ibrutinib^1^	**BTK**	0.29	**HER2**	16	**EGFR**	17				
acalabrutinib^1^	**BTK**	33								
zanubrutinib	**BTK**	0.86	**TEC**	1.8	**BMX**	3.0	**BRK**	18	**EGFR**	28
tucatinib	**HER2**	3.3	**EGFR**	6.7	**HER4**	95				
avapritinib	**PDGFRα p.D842V**	0.25	**PDGFRα**	0.27	**KIT**	12	**KIT p.D816V**	0.25		
ripretinib	**KIT**	20	**KIT p.D816V**	3.3	**PDGFRα**	5.4	**PDGFRα p.D842V**	14		
dabrafenib	**BRAF p.V600E**	1.1	**BRAF**	2.4	**RAF1**	0.57				
encorafenib	**BRAF p.V600E**	3.1	**BRAF**	7.7	**RAF1**	1.5				
vemurafenib	**BRAF p.V600E**	20	**BRAF**	31	**RAF1**	22				
binimetinib	**MEK1**	503	**MEK2**	>1000						
cobimetinib	**MEK1**	61	**MEK2**	190						
selumetinib	**MEK1**	284	**MEK2**	>1000						
trametinib	**MEK1**	11	**MEK2**	48						
alpelisib^4^	**PI3Kα**	1.4	**PI3Kγ**	9.8	**PI3Kδ**	10				

^1^Data from Uitdehaag et al. (2019) Mol Cancer Ther 18:470-481.

^2^Infigratinib received market approval in 2021.

^3^Data from Uitdehaag et al. (2014) PLoS ONE 9:e92146.

^4^Data from binding assay using SPR, as determined by Willemsen-Seegers et al. (2017) J Mol Biol 429:574-586. Values indicate K_D_ in nmol/L.

Inhibitors are ordered as discussed in this work.

All kinase inhibitors were also profiled on a panel of 134 cancer cell lines in viability assays (**Table S4**). The cell lines in the panel represent a wide range of solid tumors and hematological malignancies, including cell lines that represent relatively small patient populations, such as *FLT3* mutant and *NTRK* fusion-positive malignancies, which are targeted by some of the recently approved inhibitors (**Table 1** **;** **Table S2**).

**Figure 1:**
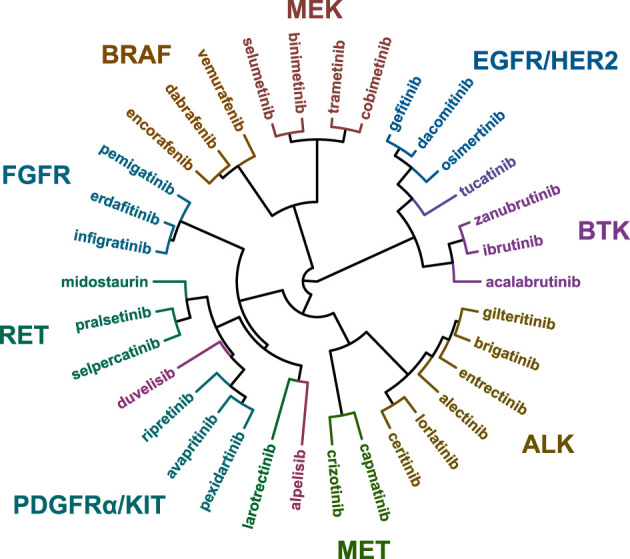
Hierarchical clustering based on the IC_50_ fingerprints of 21 recently approved kinase inhibitors and 13 previously approved drugs acting on the same targets, as determined in viability assays with 134 cancer cell lines.

The IC_50_ values of each kinase inhibitor were determined in viability assays with the 134 different cell lines in 9-point dose-response curves (**Table S4**). To determine similarities or differences in the biochemical mechanism of action of the inhibitors, the IC_50_ fingerprints of the 34 kinase inhibitors on the panel of 134 cell lines were compared by hierarchical clustering (**Figure 1**). In this analysis, compounds that act through the same target are expected to cluster together (19).

The majority of the kinase inhibitors were clustered in proximity of inhibitors sharing the same primary target, for instance the MEK, BRAF, FGFR, and BTK inhibitors (**Figure 1**). This analysis also revealed broader clusters of inhibitors which act on different targets but which function in the same pathways. For instance the BRAF and MEK inhibitors were located in distinct, but adjacent clusters, owing to the activity of the inhibitors in *BRAF* p.V600E mutant cell lines (**Figure S1**). On the contrary, some inhibitors were clustered more closely together with inhibitors which act on a different primary target. For instance entrectinib (TRK) and gilteritinib (FLT3) were clustered together with four ALK-targeted inhibitors, even though larotrectinib (TRK) and midostaurin (FLT3) were included in the analysis. This indicates differences in biochemical and cellular inhibition profiles between inhibitors sharing the same primary target. To further explore these differences between kinase inhibitors and potentially identify novel drug response biomarkers and clinical indications, we compared the biochemical selectivity and cell line targeting of inhibitors in the different clusters.

### EGFR inhibitors

Until 2021, a total of five small molecule EGFR inhibitors have received market approval: the first-generation inhibitors gefitinib and erlotinib, the second-generation inhibitors afatinib and dacomitinib, and the third-generation inhibitor osimertinib. The second- and third-generation inhibitors have an irreversible binding mode. Both *EGFR* exon 19 in-frame deletion and p.L858R mutation are predictive biomarkers for sensitivity to EGFR inhibitors in NSCLC patients and cell lines (6) and are included in the FDA label of all inhibitors except erlotinib. The *EGFR* p.T790M mutation is the most frequently observed resistance mechanism following treatment with first-generation inhibitors, and to a lesser extent with second-generation inhibitors (43). Osimertinib was specifically designed to target this mutation (44), and is approved for treatment of patients with *EGFR* p.T790M mutation-positive NSCLC who relapse after treatment with previous generation EGFR inhibitors. To compare the biochemical and cellular inhibition profiles of different generations of EGFR inhibitors, we selected the recently approved second-generation inhibitor dacomitinib and the previously approved first- and third-generation inhibitors gefitinib and osimertinib for profiling studies.

**Figure 2:**
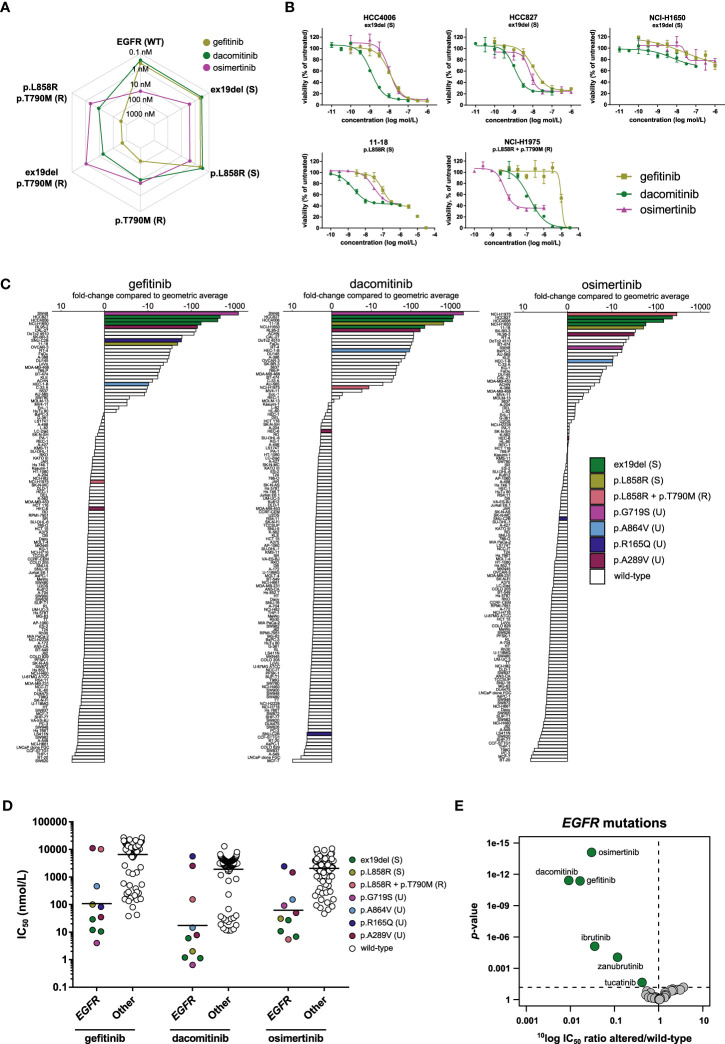
Comparison of first- (gefitinib), second- (dacomitinib), and third- (osimertinib) generation EGFR inhibitors. **(A)** Spider plot of IC_50_ values of the three inhibitors in biochemical assays with wild-type EGFR (WT) and EGFR with sensitizing (S) or resistance (R) mutations. **(B)** Dose-response curve overlays of cell viability assay results of three inhibitors in cell lines harboring sensitizing (ex19del or p.L858R) or resistance (p.L858R + p.T790M) *EGFR* mutations. **(C)** Waterfall plots of cellular responses. Cell lines harboring either sensitizing (S) or resistance (R) mutations as described in the FDA labels of the inhibitors (ex19del, p.L858R, p.T790M), or uncommon (U) *EGFR* mutations are indicated in different colors. **(D)** Scatterplots of the IC_50_ distribution of the three inhibitors across *EGFR*-mutant and wild-type cell lines. The horizontal lines indicate the geometric means. Cell lines are colored as in panel C. **(E)** Volcano plot comparing the IC_50_ differences between *EGFR*-mutant and wild-type cell lines for the 34 inhibitors. Green nodes indicate inhibitors which are significantly more active in *EGFR*-mutant cell lines compared to *EGFR* wild-type cell lines, as determined by MANOVA.

The activity of the three inhibitors in biochemical kinase assays was compared between wild-type EGFR, EGFR harboring sensitizing mutations (exon 19 deletion p.del746-750 (ex19del) and p.L858R), and EGFR harboring the p.T790M substitution which is associated with resistance (the single mutation p.T790M and the double mutants p.ex19del/T790M and p.L858R/T790M) (**Figure 2A**). The double mutants represent the clinically relevant event of an acquired resistance mutation after treatment with first- or second-generation EGFR inhibitors. In the biochemical assays, gefitinib and dacomitinib inhibited wild-type EGFR and EGFR harboring one of the sensitizing mutations with sub-nanomolar potency, while they inhibited the double mutants harboring both the p.T790M resistance mutation and a sensitizing mutation with 30- (dacomitinib) to 1000-fold (gefitinib) lower potency. Osimertinib spared wild-type EGFR and inhibited the sensitizing EGFR mutants and the mutants containing the p.T790M resistance mutation with comparable, nanomolar potency (**Figure 2A**). Compared to osimertinib, gefitinib had 171- to 435-fold lower potency on the double mutants harboring the p.T790M mutation and one of the sensitizing mutations, while dacomitinib inhibited these mutants with 4- to 17-fold lower potency compared to osimertinib (**Figure 2A**).

We next investigated whether the differences in biochemical targeting translated to a cellular context. The 134 cell line panel contained ten cell lines harboring an activating mutation in *EGFR* (**Table 3**). These include cell lines of clinical disease models for which the drugs have been approved, *e.g.*, NSCLC cell lines harboring the sensitizing ex19del (HCC4006, HCC827, NCI-H1650) or p.L858R (11–18) mutations. NCI-H1975 harbors the sensitizing p.L858R mutation in combination with the p.T790M resistance mutation (**Table 3**). Five cell lines contained point mutations other than those included in the FDA label of the three inhibitors (**Table 3**). All three inhibitors potently inhibited the viability of cell lines harboring the sensitizing mutations (**Figure 2B** **;** **Table S4**). Of note, although the potency of the three inhibitors in NCI-H1650 was comparable to their potency in the other cell lines harboring sensitizing mutations, the efficacy of all three inhibitors was marginal in this cell line (**Figure 2B**). The limited efficacy might be caused by a truncating *PTEN* mutation which renders NCI-H1650 less dependent on EGFR signaling (45).

**Table 3:** Overview of *EGFR*-mutant cell lines included in the 134 cancer cell line panel.

Cell line	Disease	*EGFR* mutation
HCC4006	Non-small cell lung cancer	Exon 19 deletion
HCC827	Non-small cell lung cancer	Exon 19 deletion
NCI-H1650	Non-small cell lung cancer	Exon 19 deletion
11-18	Non-small cell lung cancer	p.L858R
NCI-H1975	Non-small cell lung cancer	p.L858R + p.T790M
SW48	Colon adenocarcinoma	p.G719S
HEC-6	Endometrial adenocarcinoma	p.A289V
RL95-2	Endometrial adenosquamous carcinoma	p.A289V
HEC-1-B	Endometrial adenocarcinoma	p.A864V
SNU-C2B	Colon adenocarcinoma	p.R165Q

Notably, osimertinib not only inhibited the viability of cell lines harboring sensitizing *EGFR* mutations, but also potently inhibited the viability of NCI-H1975, which harbors the p.T790M resistance mutation in addition to a sensitizing mutation (**Figure 2B**). Gefitinib showed almost 1900-fold lower potency in this cell line compared to osimertinib, while dacomitinib showed 28-fold lower potency (**Figure 2B**). These results illustrate that the biochemical activity of the three generations of EGFR inhibitors translate well to a cellular context.

To determine how the response of the different *EGFR*-mutant cell lines compared to the rest of the panel, waterfall plots were generated with the IC_50_ values of the three inhibitors (**Figure 2C**). For gefitinib and dacomitinib, the most sensitive cell line had a more than 1000-fold lower IC_50_ compared to the panel average. For osimertinib this difference was around 300-fold (**Figure 2C**). Cell lines harboring the sensitizing p.L858R or ex19del mutations were among the most sensitive for all three inhibitors (**Figure 2C**), except NCI-H1975, which harbors both the p.L858R sensitizing mutation and the p.T790M resistance mutation. This was the most sensitive cell line to osimertinib, but ranked lower for dacomitinib and had an IC_50_ below average for gefitinib (**Figure 2C**), which is in line with the biochemical potency of the three inhibitors on the p.L858R/T790M mutant. The cancer cell line panel also contained five cell lines harboring mutations in EGFR at other positions, not corresponding to the clinically approved stratification markers of the three inhibitors (uncommon mutations) (**Table 3** **;** **Figures 2C, D** **)**. The colon carcinoma cell line SW48, which harbors an *EGFR* p.G719S mutation, was the most sensitive cell line for gefitinib and dacomitinib, and was also sensitive to osimertinib (**Figures 2C, D** **)**. In line with these findings, the indication of another second-generation EGFR inhibitor, afatinib, was recently expanded to also include NSCLC harboring p.G719X mutations (46). Furthermore, a phase II study is ongoing for osimertinib in patients with NSCLC harboring uncommon *EGFR* mutations, including p.G719X (NCT03434418). The endometrial carcinoma cell line RL95-2, harboring a p.A289V mutation, was among the most sensitive cell lines for all three inhibitors (**Figures 2C, D** **)**. The p.A289V mutation lies within the extracellular domain, which is frequently mutated in glioblastoma (47). Interestingly, another endometrial cell line in the panel (HEC-6) harbored the same mutation but was relatively insensitive to all three inhibitors (**Figures 2C, D** **)**, which suggests that the p.A289V mutation is not a strong predictive biomarker for sensitivity to the three EGFR inhibitors in endometrial cancers. SNU-C2B, harboring a p.R165Q mutation, which also lies in the extracellular domain, was sensitive to gefitinib, but not to dacomitinib or osimertinib, while the endometrial cell line HEC-1-B harbors a p.A864V mutation and was relatively sensitive to all three inhibitors (**Figures 2C, D** **)**.

Comparing the IC_50_ profiles of the three EGFR inhibitors between *EGFR*-mutant and wild-type cell lines shows preferential targeting of *EGFR*-mutant cell lines (**Figure 2D**). To determine whether this selective targeting was unique for EGFR inhibitors, a MANOVA was carried out for all 34 inhibitors. Although several inhibitors from different target classes significantly inhibited *EGFR*-mutant cell lines, the three EGFR inhibitors showed the strongest preference for these cell lines. Dacomitinib showed the strongest preference for targeting *EGFR-*mutant cell lines, having on average about 110-fold lower IC_50_ in *EGFR*-mutant compared to *EGFR* wild-type cell lines, followed by gefitinib (60-fold) and osimertinib (33-fold) (**Figure 2E**).

Our results show a good correlation between the biochemical and cellular potency and selectivity data for the three different generations of EGFR inhibitors. Additionally, the cell panel profiling identified several uncommon primary *EGFR* mutations which might be targeted by one or more of the currently approved EGFR inhibitors, suggesting potential room for indication expansion.

### FGFR inhibitors

FGFR is a kinase that had not been successfully targeted with small molecules until recently. The first FDA-approved FGFR inhibitor, erdafitinib, was approved for urothelial carcinoma harboring either mutations in *FGFR3* (p.R248C, p.S249C, p.G370C, and p.Y373C) or fusions involving either *FGFR2* or *FGFR3* (38). Subsequently, pemigatinib and infigratinib were both approved for *FGFR2* fusion-positive cholangiocarcinoma (39–41).

**Figure 3:**
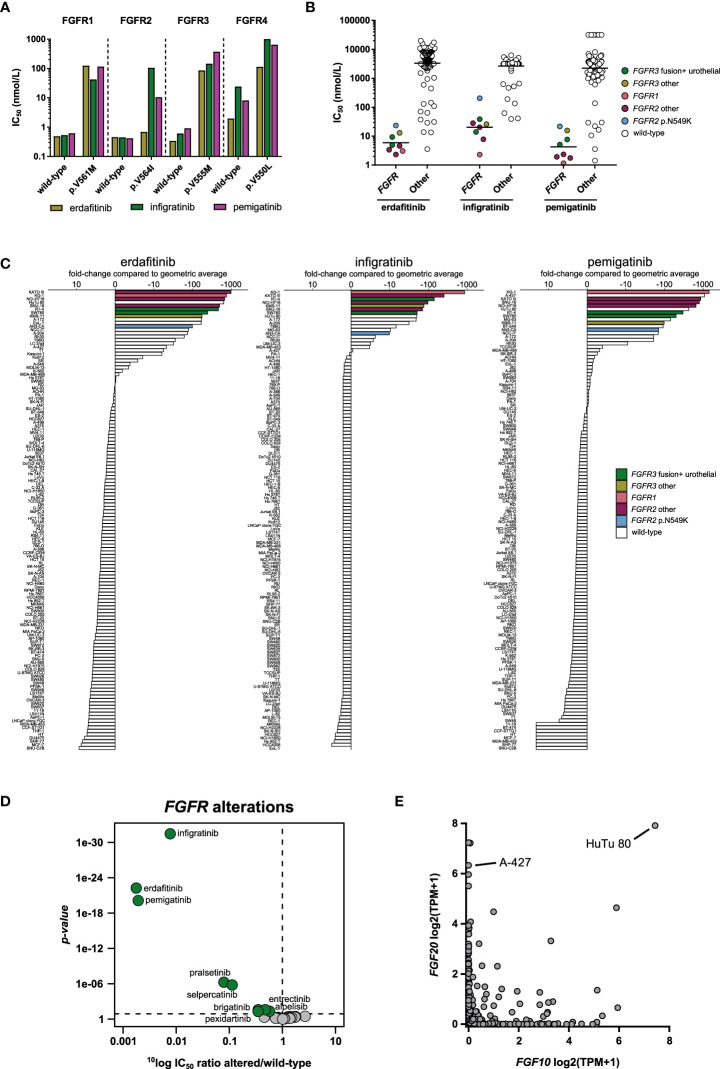
Comparison of approved FGFR inhibitors. **(A)** Bar graphs of the biochemical activity of the three FGFR inhibitors on wild-type FGFR1-4 kinases and corresponding gatekeeper mutants. **(B)** Scatterplots of the IC_50_ distribution of the three inhibitors across *FGFR*-altered and wild-type cell lines. The horizontal lines indicate the geometric means. Cell lines harboring genomic alterations in *FGFR1-3* are indicated in different colors. **(C)** Waterfall plots of cellular responses of approved FGFR inhibitors. Cell lines are colored as in panel B. **(D)** Volcano plot comparing the IC_50_ differences between *FGFR*-altered and wild-type cell lines for the 34 inhibitors. Green nodes indicate inhibitors which are significantly more potent in *FGFR*-altered cell lines compared to *FGFR* wild-type cell lines, as determined by MANOVA. **(E)** Expression of *FGF10* and *FGF20* in 1379 cell lines reported in the DepMap database. TPM, Transcripts Per Million.

In biochemical kinase assays, erdafitinib, pemigatinib and infigratinib inhibited wild-type FGFR1-3 with very similar, sub-nanomolar potencies (**Figure 3A** **;** **Table 2**). They also inhibited FGFR4, but with considerably lower potency (**Figure 3A** **;** **Table 2**). Acquired resistance mutations are a common resistance mechanism to FGFR inhibitors, in particular mutations at gatekeeper positions (48). Indeed, in our biochemical assays all three FGFR inhibitors showed lower potency on the profiled gatekeeper mutants, compared to the wild-type kinases (**Figure 3A**). Interestingly, erdafitinib retained most of its activity on the FGFR2 p.V564I mutant, suggesting that erdafitinib is still a viable treatment option in *FGFR2* p.V564I mutant cancer.

In contrast to the comparable potency between the inhibitors on wild-type FGFR1-4 in biochemical assays, the three inhibitors did show potency differences in *FGFR*-altered cell lines. The 134 cancer cell line panel included eight cell lines with an *FGFR1*-*3* alteration (either mutation, fusion, or amplification) (**Table 4**). The two bladder carcinoma cell lines RT-4 (*FGFR3*-*TACC3*) and SW780 (*FGFR3*-*BAIAP2L1*) are representative models for the therapeutic indication of erdafitinib. Due to the lack of available *FGFR2* fusion-positive cholangiocarcinoma cell lines, no representative models for the clinical indications of pemigatinib and infigratinib could be included in the study. The other six *FGFR*-altered cell lines harbored *FGFR* alterations or had a tissue origin other than those included in the indications of the three inhibitors (**Table 4**). All eight *FGFR*-altered cell lines were highly sensitive to the three inhibitors (**Figures 3B, C** **)**. Erdafitinib and pemigatinib showed comparable potency in the eight *FGFR*-altered cell lines, with average IC_50_ values of 5.9 nmol/L and 4.3 nmol/L, respectively. Infigratinib was the least potent with an average IC_50_ of 20 nmol/L, which is a more than three-fold lower potency compared to the other two inhibitors (**Figures 3B, C** **)**. Notably, the inhibitors did not show selectivity for the cell lines that are representative models for the therapeutic indication of erdafitinib (RT-4 and SW780) over the other *FGFR*-altered cell lines (**Figure 3B**). The AML cell line KG-1 harbors the *FGFR1OP2*-*FGFR1* fusion and was the most sensitive of all cell lines for infigratinib and pemigatinib, and second most sensitive for erdafitinib (**Figure 3B**). These results suggest that all three inhibitors could be beneficial for the treatment of *FGFR1*-altered malignancies. Although *FGFR1* is altered in various cancers (49), there are no kinase inhibitors approved for *FGFR1*-altered cancers yet. The gastric carcinoma cell lines KATO III and SNU-16 (*FGFR2* amplification), and the colorectal adenocarcinoma cell line NCI-H716 (*FGFR2*-*COL14A1* fusion) were also potently inhibited by all three inhibitors, with IC_50_ values ranging between 1.8 and 28 nmol/L (**Table S4**). The multiple myeloma cell line KMS-11 harbors an *FGFR3* p.Y373C mutation and has increased FGFR3 expression due to a t(4; 14) translocation (50). The IC_50_ values in this cell line ranged between 13 and 26 nmol/L (**Table S4**). Lastly, the endometrial adenocarcinoma cell line AN3-CA was the least sensitive of the *FGFR*-altered cell lines for all three inhibitors (**Figures 3B, C** **)**. This cell line harbors an *FGFR2* p.N549K mutation, which renders the kinase in an active state by disrupting the autoinhibitory function of the molecular brake (51). This mutation was identified in several *FGFR2* fusion-positive cholangiocarcinoma patients who progressed after treatment with infigratinib (52). All three inhibitors bind the inactive conformation of FGFR (53, 54), which might explain their relatively modest potency on the AN3-CA cell line compared to the other *FGFR*-altered cell lines.

**Table 4:** Overview of *FGFR*-altered cell lines included in the 134 cancer cell line panel.

Cell line	Disease	*FGFR* alteration
AN3-CA	Endometrial adenocarcinoma	*FGFR2* p.N549K
KATO III	Gastric signet ring cell adenocarcinoma	*FGFR2* amplification
KG-1	Acute myelogenous leukemia	*FGFR1OP2*-*FGFR1*
KMS-11	Multiple myeloma	*FGFR3* p.Y373C
NCI-H716	Colorectal adenocarcinoma	*FGFR2*-*COL14A1*
RT-4	Bladder transitional cell carcinoma	*FGFR3*-*TACC3*
SNU-16	Gastric adenocarcinoma	*FGFR2* amplification
SW780	Bladder transitional cell carcinoma	*FGFR3*-*BAIAP2L1*

The three inhibitors preferentially targeted *FGFR-*altered cell lines over *FGFR* wild-type cell lines in the cell line viability assays (**Figure 3C**). A MANOVA was carried out to compare the targeting of cell lines harboring *FGFR* genetic alterations by all 34 profiled kinase inhibitors. Erdafitinib and pemigatinib showed the strongest targeting of all inhibitors (**Figure 3D**). Both were, on average, over 500-fold more potent in *FGFR-*altered cell lines in comparison to wild-type cell lines, while infigratinib showed 130-fold higher potency in *FGFR-*altered compared to *FGFR* wild-type cell lines. Several other kinase inhibitors also showed significant, although lower, preference for cell lines with genetic alterations in *FGFR1-3*, including the two RET inhibitors pralsetinib and selpercatinib, which were around ten times more active in *FGFR-*altered *versus* wild-type cell lines (**Figure 3D**). The RET inhibitors cross-reacted with FGFRs in biochemical assays, likely explaining their activity in *FGFR*-altered cell lines (**Table 2**).

Among the cell lines most sensitive to FGFR inhibitors were also cell lines that were wild-type for *FGFR1-4*, e.g., HuTu 80 and A-427 (**Figure 3B** **;** **Table S4**). To identify potential drug response biomarkers in these *FGFR* wild-type cell lines, we analyzed the basal expression levels of more than 19,000 genes in the DepMap database, which contains gene expression data of 1379 cell lines. This revealed that the genes encoding the FGFR ligands FGF10 and FGF20 were highly expressed in HuTu 80 while *FGF20* was also highly expressed in A-427 (**Figure 3E**). Proliferation of *FGFR* wild-type cell lines may thus be driven by autocrine activation of FGFRs by FGFR ligands, rendering them responsive to FGFR inhibition. Besides genomic alterations in *FGFR1-3*, aberrant expression of FGFR ligands may thus be an additional response biomarker for FGFR inhibitors. Overall, our results show that the three FGFR inhibitors effectively targeted cell lines of diverse lineages which harbor distinct *FGFR* alterations or have aberrant expression of FGFR ligands, suggesting room for indication expansion and potentially a tissue-agnostic indication.

### ALK inhibitors

ALK is one of the targets for which various generations of inhibitors have been developed and approved. In 2011, the FDA approved the first-generation ALK inhibitor crizotinib, followed by the three second-generation inhibitors ceritinib, alectinib, and brigatinib in later years. The second-generation inhibitors were developed to be effective against resistance mutations acquired after crizotinib treatment, and to have improved selectivity and brain penetration (55). Most recently, the third-generation inhibitor lorlatinib was approved, which is effective against resistance mutations acquired after treatment with first- or second-generation inhibitors (55). The five ALK inhibitors were all approved for ALK-positive NSCLC and crizotinib was recently also approved for ALK-positive anaplastic large cell lymphoma (ALCL). The four second- and third-generation inhibitors cluster together based on their cellular inhibition profile, while the first-generation inhibitor crizotinib clusters with the MET inhibitor capmatinib (**Figure 1**). Crizotinib was initially developed as a MET inhibitor and consequently inhibited MET with nanomolar potency in biochemical assays, which explains the clustering with capmatinib (**Table 2**) (56). Interestingly, the ALK cluster also contains the TRK inhibitor entrectinib and the FLT3 inhibitor gilteritinib (**Figure 1**). Both inhibitors inhibited ALK biochemically with nanomolar potency, explaining their clustering with ALK inhibitors (**Table 2**).

**Figure 4:**
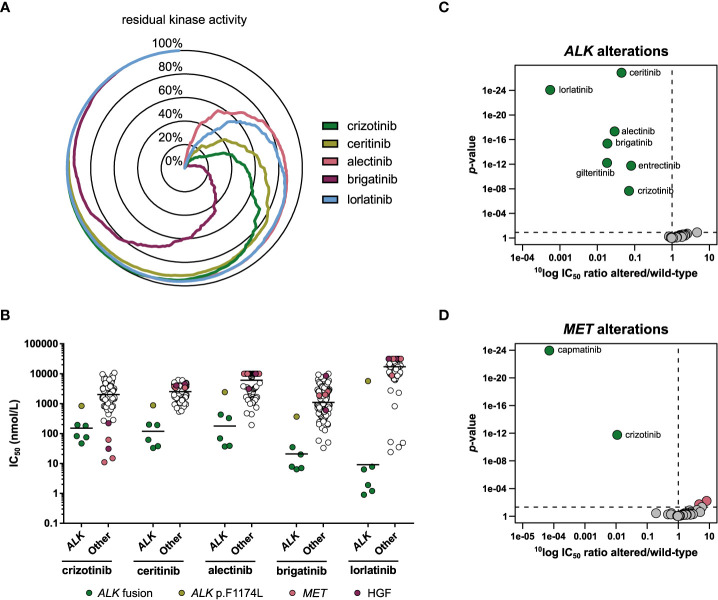
Comparison of approved ALK inhibitors. **(A)** Radar chart of the percentage residual activity of 255 wild-type kinases in the presence of 1 µmol/L inhibitor. **(B)** Scatterplots of the IC_50_ distribution of the three inhibitors across *ALK-*altered (*ALK* fusion or mutation) and *ALK* wild-type cell lines. The horizontal lines indicate the geometric means. Cell lines harboring *ALK* fusions, an *ALK* gene mutation, a *MET* gene alteration, or autocrine expression of HGF are indicated in different colors. **(C)** Volcano plot comparing the IC_50_ difference between *ALK*-altered and wild-type cell lines for 34 inhibitors. Green nodes indicate inhibitors which are significantly more potent in *ALK*-altered cell lines compared to *ALK* wild-type cell lines, as determined by MANOVA. **(D)** As panel C, but for *MET*-altered cell lines. Red nodes indicate inhibitors which are significantly less potent in *MET*-altered cell lines.

The biochemical selectivity of the ALK inhibitors was compared by profiling 255 wild-type kinases at a single concentration of 1 µmol/L inhibitor. The percentage residual activity of the kinases was compared, showing that alectinib was the most selective of the five profiled ALK inhibitors, closely followed by lorlatinib, while brigatinib was the least selective of the set (**Figure 4A**; **Table S3**).

We determined how these differences in biochemical selectivity translated to a cellular context. The cell line panel contained one NSCLC (NCI-H2228) and four ALCL cell lines (DEL, L-82, SR, SU-DHL-1) harboring an *ALK* gene fusion (**Table 5**). One cell line, the neuroblastoma cell line SK-N-SH, harbored a missense mutation (p.F1174L) in *ALK*. This mutation is frequently observed as a primary mutation in neuroblastoma (57), and in NSCLC as a secondary resistance mutation to crizotinib and ceritinib (58), but is not yet included in the FDA label of any of the ALK inhibitors. The *ALK* fusion-positive cell lines were among the most sensitive cell lines for all five ALK inhibitors (**Figure 4B**). SK-N-SH had lower sensitivity than *ALK* fusion-positive cell lines, but was still more sensitive than average (**Figure 4B**). Interestingly, lorlatinib was the most potent ALK inhibitor in the *ALK* fusion-positive cell lines, but the least potent in the p.F1174L mutant cell line (**Figure 4B**). The relatively modest activity of all ALK inhibitors in SK-N-SH may be attributed to a co-occurring oncogenic *NRAS* mutation (p.Q61K). This mutation is associated with lorlatinib resistance in both ALK-positive NSCLC and *ALK*-mutant neuroblastoma and may bypass ALK inhibition by reactivating MEK/ERK signaling (59–61). For crizotinib, the *MET*-altered cell lines and those that express the MET ligand HGF in an autocrine loop were the most sensitive instead of the *ALK* fusion-positive cell lines (**Figure 4B**), which is in line with its potent activity on MET in biochemical assays (**Table 2**).

**Table 5:** Overview of *ALK*-altered cell lines included in the 134 cancer cell line panel.

Cell line	Disease	*ALK* alteration
NCI-H2228	Non-small cell lung cancer	*EML4*-*ALK*
DEL	Anaplastic large cell lymphoma	*NPM1*-*ALK*
L-82	Anaplastic large cell lymphoma	*NPM1*-*ALK*
SR	Anaplastic large cell lymphoma	*NPM1*-*ALK*
SU-DHL-1	Anaplastic large cell lymphoma	*NPM1*-*ALK*
SK-N-SH	Neuroblastoma	p.F1174L

To determine whether any of the 34 kinase inhibitors were significantly more potent in the *ALK*-altered cell lines compared to the *ALK* wild-type cell lines, a MANOVA was carried out. This showed that all five ALK inhibitors were significantly more potent in the *ALK*-altered cell lines (**Figure 4C**). Lorlatinib showed the strongest preference with approximately 1800-fold difference in IC_50_ between mutant and wild-type cell lines, while crizotinib showed the weakest preference (14-fold) (**Figure 4C**). Entrectinib (12-fold) and gilteritinib (55-fold) also showed significant preference for inhibiting the viability of *ALK*-altered cell lines. Although not approved for ALK-related indications, entrectinib has been investigated in clinical trials involving ALK-positive malignancies (NCT03066661, NCT02568267). For gilteritinib, potential application in lorlatinib-resistant cancer has been proposed (62). In addition to the preferential targeting of *ALK*-altered cell lines by crizotinib, *MET*-altered cell lines were also significantly more responsive to crizotinib compared to *MET* wild-type cell lines (**Figure 4D**). However, whereas crizotinib was almost 100-fold more potent in *MET*-altered compared to *MET* wild-type cell lines, the selective MET inhibitor capmatinib was over 10,000-fold more potent (**Figure 4D**). These results underscore the selectivity and potency improvements achieved for ALK and MET inhibitors in the last decade. While a plethora of mutations can induce resistance to currently approved ALK inhibitors, entrectinib and gilteritinib may be further explored for expansion to indications involving specific *ALK* mutations.

### TRK inhibitors

In 2018, larotrectinib was approved for the treatment of adult and pediatric solid tumors harboring fusions of any of the three *NTRK* genes (*NTRK1-3*) (42). Roughly a year after the approval of larotrectinib, entrectinib was approved for the same tissue-agnostic indication and the additional indication of ROS1-positive NSCLC (63). *NTRK* gene fusions are oncogenic drivers in a wide variety of rare adult and pediatric tumors, where they occur at high frequencies of over 90% in some cancer types (64). On the other hand, *NTRK* gene fusions are rare in more common tumor types, such as lung- and colorectal cancer, where they occur at frequencies lower than 1% (64).

**Figure 5:**
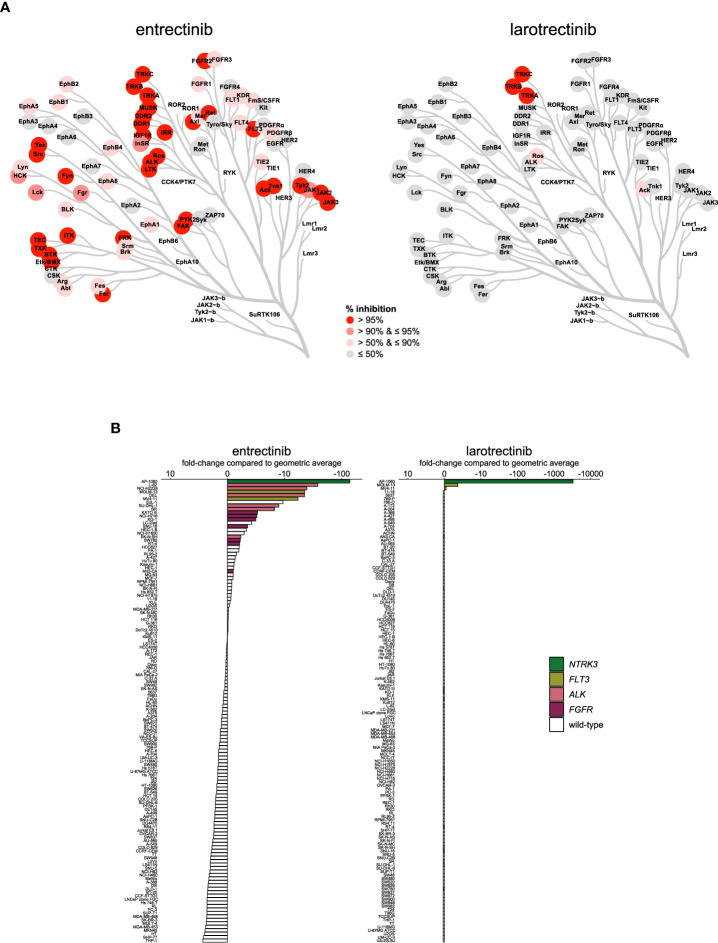
Comparison of approved TRK inhibitors. **(A)** Kinome trees showing the percentage of kinase activity inhibition for 76 wild-type tyrosine kinases in the presence of 1 µmol/L inhibitor. Presented kinases are a subset of the panel of 255 kinases as reported in Table S3. The degree of inhibition is shown from grey (least inhibited) to red (most strongly inhibited). **(B)** Waterfall plots of cellular responses of approved TRK inhibitors. Cell lines harboring an *NTRK3* fusion, *FLT3* ITD mutation, *ALK* alterations, or *FGFR* alterations are indicated in different colors.

The additional indication of ROS1-positive NSCLC for entrectinib suggests differences in the selectivity profiles of the two TRK inhibitors. This was confirmed in our biochemical assays, in which the TRK inhibitors had remarkably different selectivity profiles (**Figure 5A** **;** **Table S3**). Larotrectinib showed strong selectivity, only inhibiting TRKA, TRKB, TRKC, ROS1, and ACK1 by more than 50% at 1 µmol/L. In contrast, entrectinib inhibited 75 out of the 255 profiled kinases by more than 50% at the same concentration (**Figure 5A** **;** **Table S3**). Entrectinib was slightly more potent on TRKA-C, with around two-fold lower IC_50_ on all three TRK kinases compared to larotrectinib (**Table 2**). The apparent activity of larotrectinib on ROS1 is interesting, since this is a clinically approved biomarker for entrectinib. However, the relevance of the activity of larotrectinib on ROS1 is questionable, since it inhibited ROS1 with around 100-fold higher IC_50_ compared to TRKA-C, whereas entrectinib had comparable, nanomolar potency on both ROS1 and TRKA-C (**Table 2**). Additionally, entrectinib inhibited ALK with nanomolar potency, while larotrectinib did not inhibit ALK at concentrations up to 1 µmol/L, indicating additional differences in biochemical selectivity between the two inhibitors (**Table 2**).

Representative cell line models for the clinical indication of *NTRK* fusion-positive solid tumors are scarce and could not be obtained. However, an *ETV6-NTRK3* fusion-positive AML cell line (AP-1060) was included in our cell line panel (65). AP-1060 was potently inhibited by both inhibitors and was consequently the most sensitive cell line for both inhibitors (**Figure 5B**). This suggests that both entrectinib and larotrectinib could provide clinical benefit in *NTRK* fusion-positive hematological malignancies, as was proposed before (66). In contrast to the differences in biochemical potency on TRKC (encoded by *NTRK3*) (**Table 2**), larotrectinib was around four-fold more potent in AP-1060 compared to entrectinib (**Table S4**). In agreement with the selective biochemical profile of larotrectinib, it was also very selective in cellular assays. Aside from AP-1060, only the *FLT3* ITD-mutant cell lines MOLM-13 and MV4-11 showed some response to larotrectinib (**Figure 5B**), but this was negligible compared to the response of AP-1060 (more than 1000-fold higher IC_50_) (**Table S4**). On the other hand, entrectinib potently inhibited the *ALK*-altered and *FLT3* ITD-mutant cell lines and, to a lesser extent, the *FGFR*-altered cell lines (**Figure 5B**). The potency of entrectinib in the *ALK*-altered cell lines was not surprising, as it potently inhibits ALK in biochemical assays (**Table 2**), and was consequently evaluated in clinical trials for ALK-related indications (NCT03066661, NCT02568267). Entrectinib also inhibited FLT3 in biochemical assays, which likely explains the pronounced activity of entrectinib in the *FLT3* ITD-mutant AML cell lines (**Table S3**). The potency of entrectinib in the *FLT3* ITD-mutant AML cell lines was comparable to the potency in the *ALK*-altered cell lines, suggesting that it may be worthwhile to further explore entrectinib in *FLT3* ITD-mutant AML. Although the polypharmacology of entrectinib is associated with increased adverse events compared to larotrectinib, the broader selectivity profile of entrectinib opens opportunities for additional indications, such as *ROS1*- and *ALK*-related malignancies, and, as our data suggest, *FLT3* ITD-mutant AML (67).

### RET inhibitors

**Figure 6:**
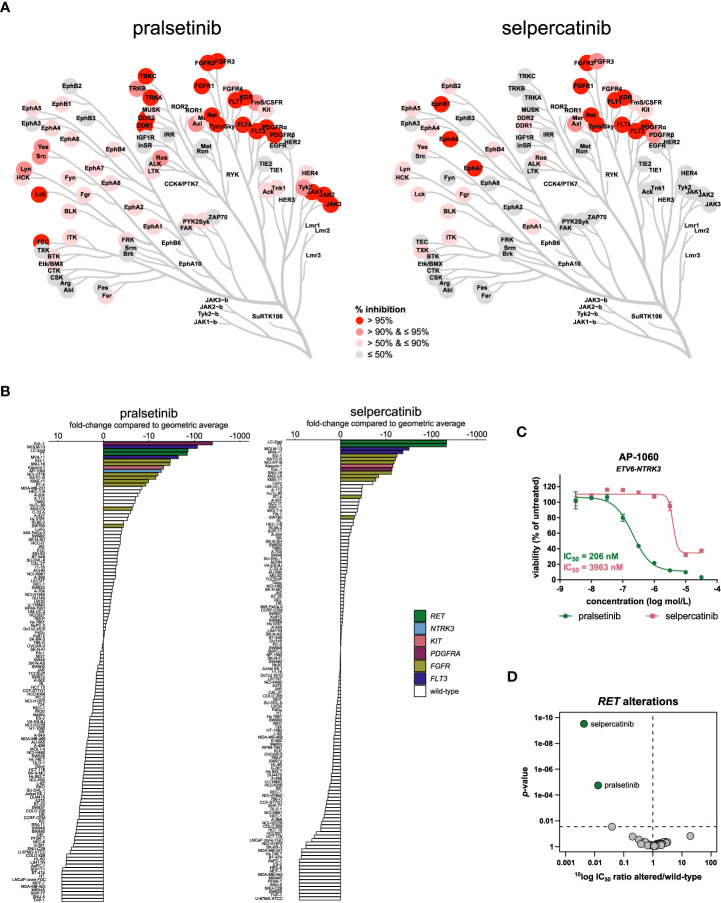
Comparison of approved RET inhibitors. **(A)** Kinome trees showing the percentage of kinase activity inhibition for 76 wild-type tyrosine kinases in the presence of 1 µmol/L inhibitor. Presented kinases are a subset of the panel of 255 kinases as reported in Table S3. The degree of inhibition is shown from grey (least inhibited) to red (most strongly inhibited). **(B)** Waterfall plots of cellular responses of approved RET inhibitors. Cell lines harboring a *RET* alteration, *NTRK3* fusion, *KIT* mutation, *PDGFRA* fusion, *FGFR* alteration, or *FLT3* ITD mutation are indicated in different colors. **(C)** Dose-response curve overlays of cell viability assay results of the RET inhibitors in the *ETV6-NTRK3* fusion-positive cell line AP-1060. **(D)** Volcano plot comparing the IC_50_ difference between *RET*-altered and *RET* wild-type cell lines for the 34 inhibitors. Green nodes indicate inhibitors which are significantly more potent in *RET*-altered cell lines compared to *RET* wild-type cell lines, as determined by MANOVA.

Selective targeting of RET is another mechanism addressed for the first time recently. Pralsetinib and selpercatinib were approved for NSCLC and thyroid cancers harboring *RET* gene fusions or mutations. Both inhibitors are investigated in tissue-agnostic clinical trials (NCT03155620, NCT03906331, NCT04589845). The inhibitors showed sub-nanomolar potency on RET in biochemical assays, but selpercatinib was almost two-fold more potent on RET compared to pralsetinib (**Table 2**). Although both inhibitors showed cross-reactivity with FGFR1-3, selectivity for RET was 10- to 100-fold (**Table 2**). Selpercatinib had a slightly more selective biochemical inhibition profile, inhibiting 46 kinases by more than 50% at 1 µmol/L whereas pralsetinib inhibited 71 kinases by more than 50% (**Figure 6A** **;** **Table S3**). In the comparative analysis of cellular inhibition profiles, the two inhibitors formed a separate cluster which, interestingly, also included the FLT3 inhibitor midostaurin (**Figure 1**). FLT3 is one of the kinases which was strongly inhibited by both inhibitors (**Figure 6A**), likely explaining the clustering with midostaurin.

The cancer cell line panel contained two cell lines harboring a *RET* gene fusion or mutation: the NSCLC cell line LC-2/ad (*CCDC6*-*RET*) and the thyroid carcinoma cell line TT (p.C634W). These cell lines are representative models for the therapeutic indications of the RET inhibitors and were among the most sensitive of the complete panel for both inhibitors (**Figure 6B**). In agreement with the higher biochemical potency of selpercatinib on RET, selpercatinib was three-fold more potent in the *RET*-altered cell lines compared to pralsetinib (**Table S4**). In addition to the *RET*-altered cell lines, both inhibitors preferentially targeted the two *FLT3* ITD-mutant AML cell lines MOLM-13 and MV4-11 (**Figure 6B**), which is in agreement with the biochemical activity on FLT3 (**Figure 6A**). Other cell lines sensitive to both inhibitors included the chronic eosinophilic leukemia (CEL) cell line EoL-1, which harbors the *FIP1L1*-*PDGFRA* fusion, the *KIT* p.N822K mutant AML cell line Kasumi-1 and the various *FGFR*-altered cell lines (**Figure 6B**). The *ETV6*-*NTRK3* fusion-positive cell line AP-1060 was almost 20-fold more sensitive to pralsetinib compared to selpercatinib (**Figure 6C**), which is in agreement with the biochemical inhibition of TRKC (encoded by *NTRK3*) by pralsetinib (**Figure 6A**). This shows that subtle selectivity differences between kinase inhibitors can be picked up by cell line profiling. Of the 34 profiled inhibitors, only the RET inhibitors showed significant preference for targeting the *RET*-altered cell lines, with a 232-fold lower IC_50_ of selpercatinib in *RET*-altered *versus* wild-type cell lines, and 76-fold lower IC_50_ of pralsetinib (**Figure 6D**). This difference in preferential targeting of *RET*-altered cell lines is consistent with the slightly higher selectivity and potency of selpercatinib in biochemical and cellular assays, which further confirms that the biochemical characteristics of the RET inhibitors translate well to a cellular context.

### FLT3 inhibitors

Gilteritinib is the second targeted inhibitor approved for treatment of AML with *FLT3* mutations, after midostaurin (68). While gilteritinib clusters together with the ALK inhibitors based on its cellular inhibition profile, midostaurin is at the other side of the clustering wheel next to the RET inhibitors (**Figure 1**).

**Figure 7:**
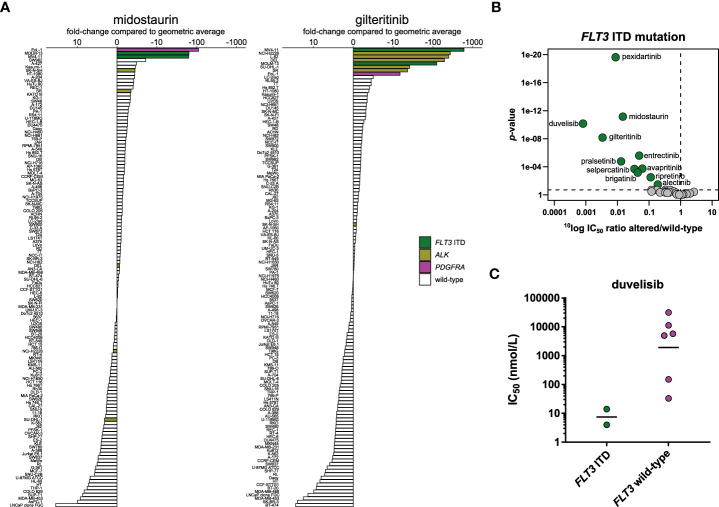
Comparison of approved FLT3 inhibitors and biomarker analysis of duvelisib. **(A)** Waterfall plots of cellular responses of approved FLT3 inhibitors. Cell lines harboring a *FLT3* ITD mutation, *PDGFRA* fusion, or *ALK* alterations are indicated in different colors. **(B)** Volcano plot comparing the IC_50_ difference between *FLT3* ITD-mutant and wild-type cell lines for the 34 inhibitors. Green nodes indicate inhibitors which are significantly more potent in *FLT3* ITD-mutant cell lines compared to *FLT3* wild-type cell lines, as determined by MANOVA. **(C)** Scatterplot of the IC_50_ distribution of the PI3Kγ/δ inhibitor duvelisib across *FLT3* ITD-mutant AML and *FLT3* wild-type AML cell lines. The horizontal lines indicate the geometric means.

In biochemical assays, gilteritinib was around eight-fold more potent on FLT3 compared to midostaurin (**Table 2**). To determine whether this difference in biochemical potency translated to a cellular setting, we profiled the inhibitors on the panel of 134 cancer cell lines. The cell line panel contained two *FLT3*-altered cell lines: the AML cell lines MV4-11 and MOLM-13. Both harbor an internal tandem duplication in *FLT3* (*FLT3* ITD), which results in constitutive, ligand-independent activation of the receptor. Gilteritinib was, on average, eight times more potent in the *FLT3* ITD-mutant cell lines compared to midostaurin, which is in agreement with the difference in biochemical potency. Unsurprisingly, the *FLT3* ITD-mutant cell lines were among the most sensitive for both inhibitors (**Figure 7A**). Additionally, both inhibitors potently inhibited the viability of the *FIP1L1*-*PDGFRA* fusion-positive cell line EoL-1, which can be explained by direct inhibition of PDGFRα, as evidenced in biochemical assays (**Table S3**). Gilteritinib also showed remarkable activity in the *ALK*-altered cell lines, owing to its potent activity on ALK in biochemical assays (**Figure 7A** **;** **Table 2**). Midostaurin did not preferentially inhibit the *ALK*-altered cell lines, which likely explains why the FLT3 inhibitors did not cluster together based on their cellular inhibition profile (**Figure 7A** **;** **Figure 1**).

MANOVA analysis of the 34 inhibitors revealed that, besides the two FLT3 inhibitors, nine other inhibitors significantly targeted the two *FLT3* ITD-mutant cell lines, which indicates widespread off-target effects of kinase inhibitors on FLT3 (**Figure 7B**). Next to entrectinib (**Figure 5B**) and the two RET inhibitors (**Figure 6B**), the FMS inhibitor pexidartinib also significantly targeted the two *FLT3* ITD-mutant cell lines. This is not surprising, since pexidartinib has been studied in a clinical trial for treatment of *FLT3* ITD-mutant AML, based on its activity on FLT3 (69). A more surprising significant hit in the MANOVA was the phosphoinositide 3-kinase (PI3K)γ/δ inhibitor duvelisib. Duvelisib is a lipid kinase inhibitor and did not inhibit FLT3 or any of the other profiled tyrosine or serine/threonine kinases in biochemical assays (**Table S3**), indicating that the *FLT3* ITD-mutant cell lines were also sensitive to inhibition of PI3Kγ or δ. To determine whether the potent effect of duvelisib was restricted to *FLT3* ITD-mutant AML cell lines, we compared the IC_50_ distribution of duvelisib across all profiled AML cell lines. Our cell line panel contained eight AML cell lines, which showed different levels of response to duvelisib (**Figure 7C**). Although the IC_50_ difference between *FLT3* ITD-mutant and *FLT3* wild-type cell lines was not significant (*p* = 0.07), the two *FLT3* ITD-mutant cell lines were the most potently inhibited of the eight AML cell lines, suggesting that *FLT3* ITD-mutant cell lines are especially sensitive to duvelisib. This finding is in line with the observed activation of the PI3K/protein kinase B (AKT)/mammalian target of rapamycin (mTOR) pathway by constitutive FLT3 activation in *FLT3* ITD-mutant AML (70).

To determine whether the combination of a FLT3 inhibitor and duvelisib was synergistic in *FLT3* ITD-mutant AML cell lines, we exposed MOLM-13 and MV4-11 to combinations of either gilteritinib or midostaurin with duvelisib. Interestingly, the combination of gilteritinib with duvelisib was additive to weakly antagonistic in MOLM-13, whereas midostaurin and duvelisib were predominantly synergistic in this cell line (**Figure S2**). Midostaurin is biochemically less selective compared to gilteritinib (**Table S3**). The synergistic effects of midostaurin and duvelisib in MOLM-13 may thus be related to off-target effects of midostaurin, and not to selective inhibition of FLT3. In contrast, both combinations were additive in MV4-11 (**Figure S2**). The additive effects of the FLT3 inhibitors with duvelisib may be explained by partial inhibition of the PI3K/AKT/mTOR pathway by duvelisib, whereas more complete inhibition of this pathway may be required for the combined inhibition of FLT3 and the PI3K/AKT/mTOR pathway to be synergistic (70). These results indicate that profiling of kinase inhibitors as single agents on a large cell line panel may provide insight into the molecular dependencies of a specific disease, but these dependencies do not always correspond with synergistic combinations.

### BTK inhibitors

Until 2021, three BTK inhibitors (ibrutinib, acalabrutinib, and most recently, zanubrutinib) were approved for treatment of diverse B-cell leukemias (71). All three inhibitors covalently bind to the sulfhydryl group of a cysteine at position 481 in the active site of BTK, resulting in irreversible inhibition of the enzyme (71–73). In biochemical kinase assays, zanubrutinib also inhibited other kinases that harbor a cysteine in the active site, such as TEC, BMX, BRK and EGFR (**Table 2**). Some of the clinical adverse events of the first-generation BTK inhibitor ibrutinib, including rash and diarrhea, have been attributed to cross-reactivity with EGFR (73). In our biochemical assays, zanubrutinib was 33 times more selective for BTK compared to EGFR, while ibrutinib was 58 times more selective (**Table 2**). To determine whether the biochemical activity profiles translated to a cellular setting, we profiled and compared the cellular activities of the three BTK inhibitors in the 134 cancer cell line panel.

**Figure 8:**
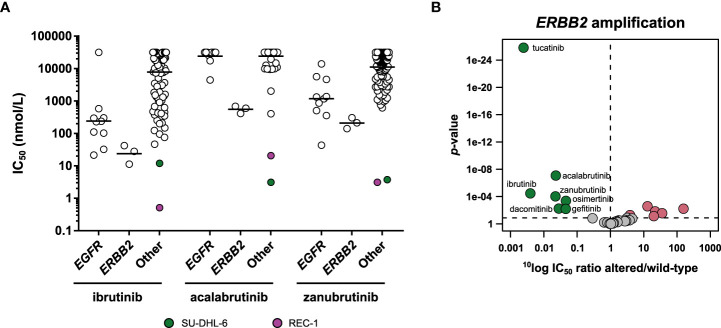
Comparison of approved BTK inhibitors. **(A)** Scatterplot of the IC_50_ distribution of the three BTK inhibitors across *EGFR*-mutant, *ERBB2*-amplified, and *EGFR* and *ERBB2* wild-type cell lines. The horizontal lines indicate the geometric means. The responsive cell lines REC-1 and SU-DHL-6 are indicated in different colors. **(B)** Volcano plot comparing the IC_50_ difference between *ERBB2*-amplified and *ERBB2* non-amplified cell lines for the 34 inhibitors. Green nodes indicate inhibitors which are significantly more potent in *ERBB2*-amplified cell lines compared to *ERBB2* non-amplified cell lines, as determined by MANOVA. Red nodes indicate inhibitors which are significantly less potent in *ERBB2*-amplified cell lines.

It should be noted that inhibition of cell viability is only a surrogate marker of *in vitro* activity of BTK inhibitors. BTK inhibitors exert their therapeutic activity by promoting egress of malignant B-cells from lymph nodes (74, 75), and inhibition of tumor cell growth is not thought to significantly contribute to their clinical efficacy. In the cell line panel, the three BTK inhibitors potently inhibited the viability of the mantle cell lymphoma cell line REC-1 and the diffuse large B-cell lymphoma (DLBCL) cell line SU-DHL-6 (**Figure 8A**). The REC-1 cell line has constitutively active B-cell receptor signaling (76). The mechanistic basis for the sensitivity of SU-DHL-6 for BTK inhibitors is unknown, but may be related to the presence of a mutation in *MYD88*. According to the DepMap database, SU-DHL-6 harbors a p.S219C mutation in *MYD88*, which is recurrently detected in primary DLBCL samples (77). Although the mutation has not been functionally characterized, BTK is a known downstream component of mutated *MYD88.* Additionally, mutated *MYD88* B-cell malignancies are known to respond to ibrutinib treatment, further suggesting that the *MYD88* p.S219C mutation underlies the sensitivity of SU-DHL-6 to BTK inhibitors (78). Ibrutinib was the most potent of the three inhibitors in the REC-1 cell line, while acalabrutinib was the most potent in SU-DHL-6 (**Figure 8A** **;** **Table S4**).

The cancer cell panel profiling also revealed cross reactivities of the three BTK inhibitors. Ibrutinib and zanubrutinib both showed significant preferential targeting of the *EGFR*-mutant cell lines, with respectively 28-fold and 8-fold higher potency in *EGFR*-mutant compared to wild-type cell lines (**Figure 2E** **;** **Table S4**). Acalabrutinib did not significantly target the *EGFR*-mutant lines. Interestingly, acalabrutinib did show preferential targeting of the three *ERBB2*- (encoding the HER2 kinase) amplified cell lines (44-fold), as did ibrutinib (254-fold) and zanubrutinib (45-fold), suggesting HER2 is a common off-target of the currently approved BTK inhibitors (**Figure 8B**). Other inhibitors which significantly targeted the *ERBB2*-amplified cell lines were the three EGFR inhibitors and the selective HER2 inhibitor tucatinib (**Figure 8B**). In previous work, we compared the cellular potency and selectivity of tucatinib to those of the other approved HER2 inhibitors lapatinib and neratinib (18). This comparison confirmed that tucatinib is the most selective of the HER2 inhibitors, while lapatinib and neratinib additionally targeted *EGFR*-mutant cell lines.

Although the BTK inhibitors showed significant off-target activities which overlapped with the targets of other profiled kinase inhibitors, they still formed a distinct cluster in the clustering wheel (**Figure 1**). This indicates that cell panel profiling can distinguish between subtle selectivity differences of kinase inhibitors.

### Inhibitors of PDGFRα and c-KIT mutants

In 2002, imatinib was the first kinase inhibitor which received approval for the treatment of KIT-positive unresectable or metastatic gastrointestinal stromal tumors (GIST). Sunitinib was approved for treatment of imatinib-resistant GIST in 2006, and in 2013 regorafenib was approved for the treatment of imatinib- and sunitinib-resistant GIST. Approximately 80% of GIST have activating mutations in *KIT* and 5-10% have activating mutations in *PDGFRA* which can be targeted by the three approved kinase inhibitors. However, approximately 10% of patients display primary resistance, and most patients that initially respond to kinase inhibitor therapy develop resistance due to secondary mutations (79). Treatment options for *KIT*- and *PDGFRA*-mutant GIST have been expanded by the recent approval of ripretinib and avapritinib. Ripretinib was developed to inhibit a broad spectrum of primary and secondary *KIT* and *PDGFRA* mutations which are not targeted by the three previously approved inhibitors (79). Avapritinib was developed to specifically target the p.D842V mutation in exon 18 of *PDGFRA*, which is the most frequent primary *PDGFRA* mutation in GIST but confers resistance to the previously approved inhibitors (80).

Avapritinib inhibited wild-type *PDGFRA* and the *PDGFRA* p.D842V mutant with sub-nanomolar potency in our biochemical assays (**Table 2**). Additionally, it had comparable nanomolar potency on the structurally similar *KIT* p.D816V mutant. This mutation is frequently observed in systemic mastocytosis, which is an additional indication for avapritinib. Ripretinib was also active on these mutants, although to a lesser extent (**Table 2**).

In line with their overlapping primary targets, the two inhibitors cluster together based on their cellular inhibition profile (**Figure 1**). No representative models for the clinical indications of the inhibitors were included in the cell line panel. However, the panel included the *FIP1L1-PDGFRA* fusion-positive CEL cell line EoL-1 and the *KIT* p.N822K mutant AML cell line Kasumi-1. Both inhibitors were most active in these cell lines, suggesting that both avapritinib and ripretinib could be beneficial for treatment of *PDGFRA*- and *KIT*-altered hematologic malignancies (**Table S4**).

**Figure 9:**
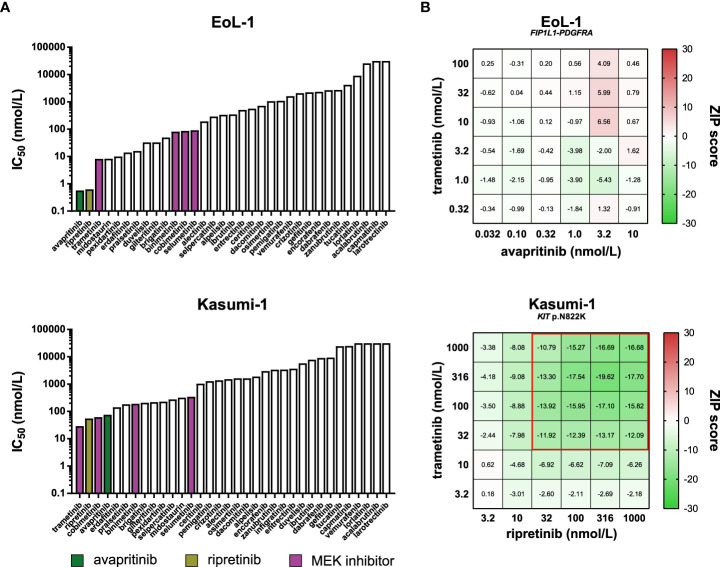
Comparison of approved PDGFRα and KIT inhibitors and combination assays. **(A)** Bar graphs presenting the IC_50_ values of the 34 kinase inhibitors in the *PDGFRA*- and *KIT*-altered cell lines EoL-1 (top) and Kasumi-1 (bottom). The PDGFRα and KIT inhibitors avapritinib and ripretinib, and the four MEK inhibitors are indicated in different colors. **(B)** Heatmaps of ZIP synergy scores for the 6x6 combination series of avapritinib and trametinib in EoL-1 (top), and ripretinib and trametinib in Kasumi-1 (bottom). The combined concentrations outlined in red are considered antagonistic (ZIP < -10).

In addition to avapritinib and ripretinib, other inhibitors potently inhibited the viability of EoL-1 and Kasumi-1 (**Figure 9A**). Many of these inhibitors targeted PDGFRα and KIT in biochemical assays, suggesting that the activity of these inhibitors in EoL-1 and Kasumi-1 was caused by off-target effects on either PDGFRα or KIT (**Table S3**). As an exception, the MEK inhibitors did not inhibit PDGFRα and KIT in biochemical assays (**Table S3**), but were still effective in EoL-1 and Kasumi-1, suggesting that EoL-1 and Kasumi-1 are also sensitive to MEK inhibition (**Figure 9A**).

Based on the dual sensitivity of EoL-1 and Kasumi-1 to PDGFRα/KIT and MEK inhibition, we sought to determine whether the combination of either avapritinib or ripretinib with a MEK inhibitor acted synergistically in these cell lines. Interestingly, all tested combinations had additive effects in EoL-1, which may indicate that EoL-1 is fully dependent on MEK signaling through the FIP1L1-PDGFRα fusion protein, and further inhibition of MEK does not provide an additional inhibitory benefit compared to inhibition of PDGFRα alone (**Figure 9B** **;** **Figure S3**) (29). Strikingly, all tested combinations were antagonistic in Kasumi-1 (**Figure 9B** **;** **Figure S3**). Full inhibition of the MEK pathway by combined KIT and MEK inhibition in Kasumi-1 may activate compensatory signaling through alternative pathways. Interestingly and in contrast to our findings, the combination of ripretinib and a MEK inhibitor was found to be synergistic in preclinical models of *KIT*-mutant GIST and systemic mastocytosis (81). Our results indicate that the synergistic effects of kinase inhibitors observed in a certain disease model cannot always be reproduced in other models, even when they harbor an activating alteration in the same driver gene. The precise molecular consequences of a genomic alteration in a specific disease should therefore be carefully determined in order to make informed decisions for drug combinations.

## Conclusions

As the spectrum of approved kinase inhibitors for oncology indications expands, it is essential to determine and compare the potency and selectivity of these inhibitors to make the most informed decisions about therapy selection and label expansion. This study provides a head-to-head comparison of 21 newly approved kinase inhibitors and 13 previously approved comparators by extensive biochemical and cell panel profiling. For 11 of the newly approved inhibitors this is the first large-scale kinase profiling study in the public domain, and for seven this is the first profiling study on a large cancer cell line panel. We find that the biochemical characteristics of these kinase inhibitors do not always translate to a cellular context, indicating that cell panel profiling can provide additional insights into kinase inhibitor activity compared to biochemical profiling alone. Lastly, the patient stratification markers currently described in the FDA labels of kinase inhibitors were confirmed to a high degree in our assays, and we additionally identified potential predictive drug response biomarkers which may warrant further investigation for label expansion of the approved kinase inhibitors.

## Data availability statement

The original contributions presented in the study are included in the article/**Supplementary Material**. Further inquiries can be directed to the corresponding author.

## Author contributions

Conceptualization, JK, GZ, and JU; Resources, GZ and YK; Investigation, JK, WR, JD, MP, YG, TB, AD, JM, JU, YN, YK, JR, NW-S, and GZ; Visualization, JK; Writing – original draft, JK and GZ; Writing – review and editing, WR, YG, and TB; All authors contributed to the article and approved the submitted version.

## Conflict of interest

GZ is managing director and shareholder of Oncolines B.V. JK, WR, JD, YG, TB, AD, JM, JR, NW-S are employees of Oncolines B.V. MP is employee of NTRC Therapeutics B.V. JU is employee of Acerta Pharma B.V., a member of the AstraZeneca Group. YN, YK are employees of Carna Biosciences, Inc.

## Publisher’s note

All claims expressed in this article are solely those of the authors and do not necessarily represent those of their affiliated organizations, or those of the publisher, the editors and the reviewers. Any product that may be evaluated in this article, or claim that may be made by its manufacturer, is not guaranteed or endorsed by the publisher.

## References

[1] Cohen P. Protein kinases–the major drug targets of the twenty-first century? Nat Rev Drug Discovery (2002) 1:309–15. doi: 10.1038/nrd773 12120282

[2] Roskoski R. Properties of FDA-approved small molecule protein kinase inhibitors: A 2020 update. Pharmacol Res (2020) 152:104609. doi: 10.1016/j.phrs.2019.104609 31862477

[3] Attwood MM Fabbro D v. SA Knapp S Schiöth HB. Trends in kinase drug discovery: targets, indications and inhibitor design. Nat Rev Drug Discovery (2021) 20:839–61. doi: 10.1038/s41573-021-00252-y 34354255

[4] Manning G Whyte DB Martinez R Hunter T Sudarsanam S. The protein kinase complement of the human genome. Science (2002) 298:1912–34. doi: 10.1126/science.1075762 12471243

[5] Druker BJ Talpaz M Resta DJ Peng B Buchdunger E Ford JM. Efficacy and safety of a specific inhibitor of the BCR-ABL tyrosine kinase in chronic myeloid leukemia. N Engl J Med (2001) 344:1031–7. doi: 10.1056/NEJM200104053441401 11287972

[6] Pao W Miller V Zakowski M Doherty J Politi K Sarkaria I. EGF receptor gene mutations are common in lung cancers from “never smokers” and are associated with sensitivity of tumors to gefitinib and erlotinib. Proc Natl Acad Sci U.S.A. (2004) 101:13306–11. doi: 10.1073/pnas.0405220101 PMC516528 15329413

[7] Yang H Higgins B Kolinsky K Packman K Go Z Iyer R. RG7204 (PLX4032), a selective BRAFV600E inhibitor, displays potent antitumor activity in preclinical melanoma models. Cancer Res (2010) 70:5518–27. doi: 10.1158/0008-5472.CAN-10-0646 20551065

[8] Kwak EL Bang Y-J Camidge DR Shaw AT Solomon B Maki RG. Anaplastic lymphoma kinase inhibition in non-small-cell lung cancer. N Engl J Med (2010) 363:1693–703. doi: 10.1056/NEJMoa1006448 PMC3014291 20979469

[9] Pao W Wang TY Riely GJ Miller VA Pan Q Ladanyi M. KRAS mutations and primary resistance of lung adenocarcinomas to gefitinib or erlotinib. PloS Med (2005) 2:e17. doi: 10.1371/journal.pmed.0020017 15696205 PMC545207

[10] Amado RG Wolf M Peeters M van Cutsem E Siena S Freeman DJ. Wild-type KRAS is required for panitumumab efficacy in patients with metastatic colorectal cancer. J Clin Oncol (2008) 26:1626–34. doi: 10.1200/JCO.2007.14.7116 18316791

[11] Karapetis CS Khambata-Ford S Jonker DJ O’Callaghan CJ Tu D Tebbutt NC. K-Ras mutations and benefit from cetuximab in advanced colorectal cancer. N Engl J Med (2008) 359:1757–65. doi: 10.1056/NEJMoa0804385 18946061

[12] Shoemaker RH. The NCI60 human tumour cell line anticancer drug screen. Nat Rev Cancer (2006) 6:813–23. doi: 10.1038/nrc1951 16990858

[13] Barretina J Caponigro G Stransky N Venkatesan K Margolin AA Kim S. The cancer cell line encyclopedia enables predictive modeling of anticancer drug sensitivity. Nature (2012) 483:603–7. doi: 10.1038/nature11003 PMC3320027 22460905

[14] Garnett MJ Edelman EJ Heidorn SJ Greenman CD Dastur A Lau KW. Systematic identification of genomic markers of drug sensitivity in cancer cells. Nature (2012) 483:570–5. doi: 10.1038/nature11005 PMC3349233 22460902

[15] Uitdehaag JCM de Roos JADM van Doornmalen AM Prinsen MBW de Man J Tanizawa Y. Comparison of the cancer gene targeting and biochemical selectivities of all targeted kinase inhibitors approved for clinical use. PloS One (2014) 9:e92146. doi: 10.1371/journal.pone.0092146 24651269 PMC3961306

[16] Ghandi M Huang FW Jané-Valbuena J v. KG CC >Lo ER M. Next-generation characterization of the cancer cell line encyclopedia. Nature (2019) 569:503–8. doi: 10.1038/s41586-019-1186-3 PMC6697103 31068700

[17] Uitdehaag JCM Kooijman JJ de Roos JADM Prinsen MBW Dylus J Willemsen-Seegers N. Combined cellular and biochemical profiling to identify predictive drug response biomarkers for kinase inhibitors approved for clinical use between 2013 and 2017. Mol Cancer Ther (2019) 18:470–81. doi: 10.1158/1535-7163.MCT-18-0877 30381447

[18] Conlon NT Kooijman JJ van Gerwen SJC Mulder WR Zaman GJR Diala I. Comparative analysis of drug response and gene profiling of HER2-targeted tyrosine kinase inhibitors. Br J Cancer (2021) 124:1249–59. doi: 10.1038/s41416-020-01257-x PMC8007737 33473169

[19] Uitdehaag JCM de Roos JADM Prinsen MBW Willemsen-Seegers N de Vetter JRF Dylus J. Cell panel profiling reveals conserved therapeutic clusters and differentiates the mechanism of action of different PI3K/mTOR, aurora kinase and EZH2 inhibitors. Mol Cancer Ther (2016) 15:3097–109. doi: 10.1158/1535-7163.MCT-16-0403 27587489

[20] Libouban MAA de Roos JADM Uitdehaag JCM Willemsen-Seegers N Mainardi S Dylus J. Stable aneuploid tumors cells are more sensitive to TTK inhibition than chromosomally unstable cell lines. Oncotarget (2017) 8:38309–25. doi: 10.18632/oncotarget.16213 PMC5503534 28415765

[21] Davis MI Hunt JP Herrgard S Ciceri P Wodicka LM Pallares G. Comprehensive analysis of kinase inhibitor selectivity. Nat Biotechnol (2011) 29:1046–51. doi: 10.1038/nbt.1990 22037378

[22] Klaeger S Heinzlmeir S Wilhelm M Polzer H Vick B Koenig P-A. The target landscape of clinical kinase drugs. Science (1979) 2017) 358:eaan4368. doi: 10.1126/science.aan4368 PMC6542668 29191878

[23] Iorio F Knijnenburg TA Vis DJ Bignell GR Menden MP Schubert M. A landscape of pharmacogenomic interactions in cancer. Cell (2016) 166:740–54. doi: 10.1016/j.cell.2016.06.017 PMC4967469 27397505

[24] Rees MG Seashore-Ludlow B Cheah JH Adams DJ v. PE Gill S. Correlating chemical sensitivity and basal gene expression reveals mechanism of action. Nat Chem Biol (2016) 12:109–16. doi: 10.1038/nchembio.1986 PMC4718762 26656090

[25] Yu C Mannan AM Yvone GM Ross KN Zhang Y-L Marton MA. High-throughput identification of genotype-specific cancer vulnerabilities in mixtures of barcoded tumor cell lines. Nat Biotechnol (2016) 34:419–23. doi: 10.1038/nbt.3460 PMC5508574 26928769

[26] Corsello SM Nagari RT Spangler RD Rossen J Kocak M Bryan JG. Discovering the anti-cancer potential of non-oncology drugs by systematic viability profiling. Nat Cancer (2020) 1:235–48. doi: 10.1038/s43018-019-0018-6 PMC7328899 32613204

[27] Kitagawa D Yokota K Gouda M Narumi Y Ohmoto H Nishiwaki E. Activity-based kinase profiling of approved tyrosine kinase inhibitors. Genes Cells (2013) 18:110–22. doi: 10.1111/gtc.12022 23279183

[28] Metz KS Deoudes EM Berginski ME Jimenez-Ruiz I Aksoy BA Hammerbacher J. Coral: Clear and customizable visualization of human kinome data. Cell Syst (2018) 7:347–350.e1. doi: 10.1016/j.cels.2018.07.001 30172842 PMC6366324

[29] Shen J Li L Yang T Cohen PS Sun G. Biphasic mathematical model of cell–drug interaction that separates target-specific and off-target inhibition and suggests potent targeted drug combinations for multi-driver colorectal cancer cells. Cancers (Basel) (2020) 12:436. doi: 10.3390/cancers12020436 PMC7072552 32069833

[30] Bairoch A. The cellosaurus, a cell-line knowledge resource. J Biomol Tech (2018) 29:25–38. doi: 10.7171/jbt.18-2902-002 29805321 PMC5945021

[31] Chang MT Bhattarai TS Schram AM Bielski CM Donoghue MTA Jonsson P. Accelerating discovery of functional mutant alleles in cancer. Cancer Discovery (2018) 8:174–83. doi: 10.1158/2159-8290.CD-17-0321 PMC5809279 29247016

[32] Miyake M Ishii M Koyama N Kawashima K Kodama T Anai S. 1-tert-Butyl-3-[6-(3,5-dimethoxy-phenyl)-2-(4-diethylamino-butylamino)-pyrido[2,3-d]pyrimidin-7-yl]-urea (PD173074), a selective tyrosine kinase inhibitor of fibroblast growth factor receptor-3 (FGFR3), inhibits cell proliferation of bladder cancer carrying the FGFR3 gene mutation along with up-regulation of p27/Kip1 and G1/G0 arrest. J Pharmacol Exp Ther (2010) 332:795–802. doi: 10.1124/jpet.109.162768 19955487

[33] Yadav B Wennerberg K Aittokallio T Tang J. Searching for drug synergy in complex dose-response landscapes using an interaction potency model. Comput Struct Biotechnol J (2015) 13:504–13. doi: 10.1016/j.csbj.2015.09.001 PMC4759128 26949479

[34] Zou HY Li Q Engstrom LD West M Appleman V Wong KA. PF-06463922 is a potent and selective next-generation ROS1/ALK inhibitor capable of blocking crizotinib-resistant ROS1 mutations. Proc Natl Acad Sci U.S.A. (2015) 112:3493–8. doi: 10.1073/pnas.1420785112 PMC4371934 25733882

[35] Engelman JA Zejnullahu K Gale CM Lifshits E Gonzales AJ Shimamura T. PF00299804, an irreversible pan-ERBB inhibitor, is effective in lung cancer models with EGFR and ERBB2 mutations that are resistant to gefitinib. Cancer Res (2007) 67:11924–32. doi: 10.1158/0008-5472.CAN-07-1885 18089823

[36] Benner B Good L Quiroga D Schultz TE Kassem M Carson WE. Pexidartinib, a novel small molecule CSF-1R inhibitor in use for tenosynovial giant cell tumor: A systematic review of pre-clinical and clinical development. Drug Des Devel Ther (2020) 14:1693–704. doi: 10.2147/DDDT.S253232 PMC7210448 32440095

[37] Liu X Wang Q Yang G Marando C Koblish HK Hall LM. A novel kinase inhibitor, INCB28060, blocks c-MET-dependent signaling, neoplastic activities, and cross-talk with EGFR and HER-3. Clin Cancer Res (2011) 17:7127–38. doi: 10.1158/1078-0432.CCR-11-1157 21918175

[38] Perera TPS Jovcheva E Mevellec L Vialard J de Lange D Verhulst T. Discovery & pharmacological characterization of JNJ-42756493 (Erdafitinib), a functionally selective small-molecule FGFR family inhibitor. Mol Cancer Ther (2017) 16:1010–20. doi: 10.1158/1535-7163.MCT-16-0589 28341788

[39] Liu PCC Koblish H Wu L Bowman K Diamond S DiMatteo D. INCB054828 (pemigatinib), a potent and selective inhibitor of fibroblast growth factor receptors 1, 2, and 3, displays activity against genetically defined tumor models. PloS One (2020) 15:e0231877. doi: 10.1371/journal.pone.0231877 32315352 PMC7313537

[40] Guagnano V Furet P Spanka C Bordas V le Douget M Stamm C. Discovery of 3-(2,6-Dichloro-3,5-dimethoxy-phenyl)-1-{6-[4-(4-ethyl- piperazin-1-yl)-phenylamino]-pyrimidin-4-yl}-1-methyl-urea (NVP-BGJ398), a potent and selective inhibitor of the fibroblast growth factor receptor family of receptor tyrosine kinase. J Med Chem (2011) 54:7066–83. doi: 10.1021/jm2006222 21936542

[41] Guagnano V Kauffmann A Wöhrle S Stamm C Ito M Barys L. FGFR genetic alterations predict for sensitivity to NVP-BGJ398, a selective pan-FGFR inhibitor. Cancer Discovery (2012) 2:1118–33. doi: 10.1158/2159-8290.CD-12-0210 23002168

[42] Drilon A Laetsch TW Kummar S DuBois SG Lassen UN Demetri GD. Efficacy of larotrectinib in TRK fusion–positive cancers in adults and children. N Engl J Med (2018) 378:731–9. doi: 10.1056/NEJMoa1714448 PMC5857389 29466156

[43] Westover D Zugazagoitia J Cho BC Lovly CM Paz-Ares L. Mechanisms of acquired resistance to first- and second-generation EGFR tyrosine kinase inhibitors. Ann Oncol (2018) 29:i10–9. doi: 10.1093/annonc/mdx703 PMC6454547 29462254

[44] Cross DAE Ashton SE Ghiorghiu S Eberlein C Nebhan CA Spitzler PJ. AZD9291, an irreversible EGFR TKI, overcomes T790M-mediated resistance to EGFR inhibitors in lung cancer. Cancer Discovery (2014) 4:1046–61. doi: 10.1158/2159-8290.CD-14-0337 PMC4315625 24893891

[45] Ayestaran I Galhoz A Mcdermott U Iorio F Menden Correspondence MP. Identification of intrinsic drug resistance and its biomarkers in high-throughput pharmacogenomic and CRISPR screens. Patterns (2020) 1:100065. doi: 10.1016/j.patter.2020.100065 33205120 PMC7660407

[46] Yang JCH v. SL SL G CM T Mok TSK Schuler M. Clinical activity of afatinib in patients with advanced non-small-cell lung cancer harbouring uncommon EGFR mutations: a combined post-hoc analysis of LUX-lung 2, LUX-lung 3, and LUX-lung 6. Lancet Oncol (2015) 16:830–8. doi: 10.1016/S1470-2045(15)00026-1 26051236

[47] Binder ZA Thorne AH Bakas S Wileyto EP Bilello M Akbari H. Epidermal growth factor receptor extracellular domain mutations in glioblastoma present opportunities for clinical imaging and therapeutic development. Cancer Cell (2018) 34:163–177.e7. doi: 10.1016/j.ccell.2018.06.006 29990498 PMC6424337

[48] Yue S Li Y Chen X Wang J Li M Chen Y. FGFR-TKI resistance in cancer: current status and perspectives. J Hematol Oncol (2021) 14:23. doi: 10.1186/s13045-021-01040-2 33568192 PMC7876795

[49] Ho HK Yeo AHL Kang TS Chua BT. Current strategies for inhibiting FGFR activities in clinical applications: opportunities, challenges and toxicological considerations. Drug Discovery Today (2014) 19:51–62. doi: 10.1016/j.drudis.2013.07.021 23932951

[50] Kalff A Spencer A. The t (4,14) translocation and FGFR3 overexpression in multiple myeloma: prognostic implications and current clinical strategies. Blood Cancer J (2012) 2:e89. doi: 10.1038/bcj.2012.37 22961061 PMC3461707

[51] Farrell B Breeze AL. Structure, activation and dysregulation of fibroblast growth factor receptor kinases: perspectives for clinical targeting. Biochem Soc Trans (2018) 46:1753–70. doi: 10.1042/BST20180004 PMC6299260 30545934

[52] Goyal L Saha SK Liu LY Siravegna G Leshchiner I Ahronian LG. Polyclonal secondary FGFR2 mutations drive acquired resistance to FGFR inhibition in patients with FGFR2 fusion-positive cholangiocarcinoma. Cancer Discovery (2017) 7:252–63. doi: 10.1158/2159-8290.CD-16-1000 PMC5433349 28034880

[53] Roskoski R. The role of fibroblast growth factor receptor (FGFR) protein-tyrosine kinase inhibitors in the treatment of cancers including those of the urinary bladder. Pharmacol Res (2020) 151:104567. doi: 10.1016/j.phrs.2019.104567 31770593

[54] Lin Q Chen X Qu L Guo M Wei H Dai S. Characterization of the cholangiocarcinoma drug pemigatinib against FGFR gatekeeper mutants. Commun Chem (2022) 5:100. doi: 10.1038/s42004-022-00718-z PMC9814635 36698015

[55] Spagnuolo A Maione P Gridelli C. Evolution in the treatment landscape of non-small cell lung cancer with ALK gene alterations: from the first- to third-generation of ALK inhibitors. Expert Opin Emerg Drugs (2018) 23:231–41. doi: 10.1080/14728214.2018.1527902 30251885

[56] Zou HY Li Q Lee JH Arango ME McDonnell SR Yamazaki S. An orally available small-molecule inhibitor of c-met, PF-2341066, exhibits cytoreductive antitumor efficacy through antiproliferative and antiangiogenic mechanisms. Cancer Res (2007) 67:4408–17. doi: 10.1158/0008-5472.CAN-06-4443 17483355

[57] Mossé YP Laudenslager M Longo L Cole KA Wood A Attiyeh EF. Identification of ALK as a major familial neuroblastoma predisposition gene. Nature (2008) 455:930–5. doi: 10.1038/nature07261 PMC2672043 18724359

[58] Pan Y Deng C Qiu Z Cao C Wu F. The resistance mechanisms and treatment strategies for ALK-rearranged non-small cell lung cancer. Front Oncol (2021) 11:713530. doi: 10.3389/fonc.2021.713530 34660278 PMC8517331

[59] Yoda S Lin JJ Lawrence MS Burke BJ Friboulet L Langenbucher A. Sequential ALK inhibitors can select for lorlatinib-resistant compound ALK mutations in ALK-positive lung cancer. Cancer Discovery (2018) 8:714–29. doi: 10.1158/2159-8290.CD-17-1256 PMC5984716 29650534

[60] Liu T Merguerian MD Rowe SP Pratilas CA Chen AR Ladle BH. Exceptional response to the ALK and ROS1 inhibitor lorlatinib and subsequent mechanism of resistance in relapsed ALK F1174L-mutated neuroblastoma. Cold Spring Harb Mol Case Stud (2021) 7:a006064. doi: 10.1101/mcs.a006064 34210658 PMC8327881

[61] George RE Sanda T Hanna M Fröhling S Luther W Zhang J. Activating mutations in ALK provide a therapeutic target in neuroblastoma. Nature (2008) 455:975–8. doi: 10.1038/nature07397 PMC2587486 18923525

[62] Mizuta H Okada K Araki M Adachi J Takemoto A Kutkowska J. Gilteritinib overcomes lorlatinib resistance in ALK-rearranged cancer. Nat Commun (2021) 12:1261. doi: 10.1038/s41467-021-21396-w 33627640 PMC7904790

[63] Menichincheri M Ardini E Magnaghi P Avanzi N Banfi P Bossi R. Discovery of entrectinib: A new 3-aminoindazole as a potent anaplastic lymphoma kinase (ALK), c-ros oncogene 1 kinase (ROS1), and pan-tropomyosin receptor kinases (Pan-TRKs) inhibitor. J Med Chem (2016) 59:3392–408. doi: 10.1021/acs.jmedchem.6b00064 27003761

[64] Cocco E Scaltriti M Drilon A. NTRK fusion-positive cancers and TRK inhibitor therapy. Nat Rev Clin Oncol (2018) 15:731–47. doi: 10.1038/s41571-018-0113-0 PMC6419506 30333516

[65] Chen S Nagel S Schneider B Dai H Geffers R Kaufmann M. A new ETV6-NTRK3 cell line model reveals MALAT1 as a novel therapeutic target - a short report. Cell Oncol (Dordr) (2018) 41:93–101. doi: 10.1007/s13402-017-0356-2 29119387 PMC12995242

[66] Joshi SK Davare MA Druker BJ Tognon CE. Revisiting NTRKs as an emerging oncogene in hematological malignancies. Leukemia (2019) 33:2563–74. doi: 10.1038/s41375-019-0576-8 PMC7410820 31551508

[67] Dunn DB. Larotrectinib and entrectinib: TRK inhibitors for the treatment of pediatric and adult patients with NTRK gene fusion. J Adv Pract Oncol (2020) 11:418–23. doi: 10.6004/jadpro.2020.11.4.9 PMC7863124 33604102

[68] Mori M Kaneko N Ueno Y Yamada M Tanaka R Saito R. Gilteritinib, a FLT3/AXL inhibitor, shows antileukemic activity in mouse models of FLT3 mutated acute myeloid leukemia. Invest New Drugs (2017) 35:556–65. doi: 10.1007/s10637-017-0470-z PMC5613053 28516360

[69] Smith CC Levis MJ Frankfurt O Pagel JM Roboz GJ Stone RM. A phase 1/2 study of the oral FLT3 inhibitor pexidartinib in relapsed/refractory FLT3-ITD–mutant acute myeloid leukemia. Blood Adv (2020) 4:1711–21. doi: 10.1182/bloodadvances.2020001449 PMC7189289 32330242

[70] Darici S Zavatti M Braglia L Accordi B Serafin V Horne GA. Synergistic cytotoxicity of dual PI3K/mTOR and FLT3 inhibition in FLT3-ITD AML cells. Adv Biol Regul (2021) 82:100830. doi: 10.1016/j.jbior.2021.100830 34555701

[71] Guo Y Liu Y Hu N Yu D Zhou C Shi G. Discovery of zanubrutinib (BGB-3111), a novel, potent, and selective covalent inhibitor of bruton’s tyrosine kinase. J Med Chem (2019) 62:7923–40. doi: 10.1021/acs.jmedchem.9b00687 31381333

[72] Pan Z Scheerens H Li SJ Schultz BE Sprengeler PA Burrill LC. Discovery of selective irreversible inhibitors for bruton’s tyrosine kinase. ChemMedChem (2007) 2:58–61. doi: 10.1002/cmdc.200600221 17154430

[73] Barf T Covey T Izumi R van de Kar B Gulrajani M van Lith B. Acalabrutinib (ACP-196): A covalent bruton tyrosine kinase inhibitor with a differentiated selectivity and *in vivo* potency profile. J Pharmacol Exp Ther (2017) 363:240–52. doi: 10.1124/jpet.117.242909 28882879

[74] Ponader S Chen SS Buggy JJ Balakrishnan K Gandhi V Wierda WG. The bruton tyrosine kinase inhibitor PCI-32765 thwarts chronic lymphocytic leukemia cell survival and tissue homing *in vitro* and in vivo. Blood (2012) 119:1182–9. doi: 10.1182/blood-2011-10-386417 PMC4916557 22180443

[75] de Rooij MFM Kuil A Geest CR Eldering E Chang BY Buggy JJ. The clinically active BTK inhibitor PCI-32765 targets b-cell receptor– and chemokine-controlled adhesion and migration in chronic lymphocytic leukemia. Blood (2012) 119:2590–4. doi: 10.1182/blood-2011-11-390989 22279054

[76] Rahal R Frick M Romero R Korn JM Kridel R Chan FC. Pharmacological and genomic profiling identifies NF-κB-targeted treatment strategies for mantle cell lymphoma. Nat Med (2014) 20:87–92. doi: 10.1038/nm.3435 24362935

[77] Ngo VN Young RM Schmitz R Jhavar S Xiao W Lim KH. Oncogenically active MYD88 mutations in human lymphoma. Nature (2011) 470:115–9. doi: 10.1038/nature09671 PMC5024568 21179087

[78] Munshi M Liu X Chen JG Xu L Tsakmaklis N Demos MG. SYK is activated by mutated MYD88 and drives pro-survival signaling in MYD88 driven b-cell lymphomas. Blood Cancer J (2020) 10:12. doi: 10.1038/s41408-020-0277-6 32005797 PMC6994488

[79] Smith BD Kaufman MD Lu WP Gupta A Leary CB Wise SC. Ripretinib (DCC-2618) is a switch control kinase inhibitor of a broad spectrum of oncogenic and drug-resistant KIT and PDGFRA variants. Cancer Cell (2019) 35:738–751.e9. doi: 10.1016/j.ccell.2019.04.006 31085175

[80] von Mehren M Heinrich MC Shi H Iannazzo S Mankoski R Dimitrijević S. Clinical efficacy comparison of avapritinib with other tyrosine kinase inhibitors in gastrointestinal stromal tumors with PDGFRA D842V mutation: a retrospective analysis of clinical trial and real-world data. BMC Cancer (2021) 21:291. doi: 10.1186/s12885-021-08013-1 33740926 PMC7976710

[81] Gupta A Singh J García-Valverde A Serrano C Flynn DL Smith BD. Ripretinib and MEK inhibitors synergize to induce apoptosis in preclinical models of GIST and systemic mastocytosis. Mol Cancer Ther (2021) 20:1234–45. doi: 10.1158/1535-7163.MCT-20-0824 33947686

