# Supplementary material for: Comparative kinase and cancer cell panel profiling of kinase inhibitors approved for clinical use from 2018 to 2020
Source: Front Oncol. 2022 Sep 14;12:953013. doi: 10.3389/fonc.2022.953013 (PMC9516332; doi:10.3389/fonc.2022.953013)
Supplement: Supplementary file 5 [file DataSheet_1.pdf]

Supplementary Material

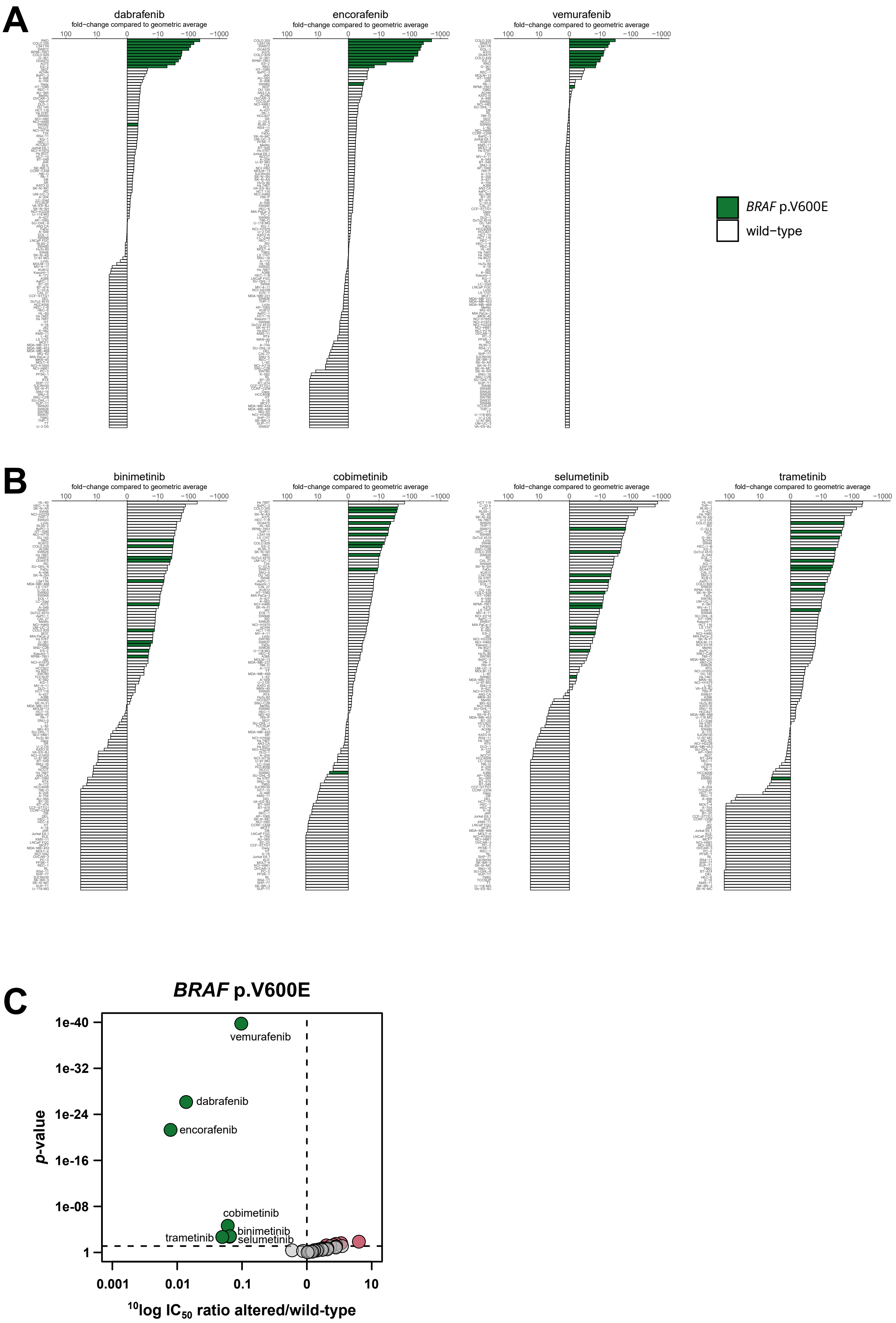

Figure S1. Comparison of approved BRAF and MEK1 inhibitors. **(A)** Waterfall plots of cellular responses of the three approved BRAF inhibitors dabrafenib, encorafenib and vemurafenib. *BRAF* p.V600E-mutant cell lines are indicated in green. **(B)** Waterfall plots of cellular responses of the four approved MEK1 inhibitors binimetinib, cobimetinib, selumetinib and trametinib. *BRAF* p.V600E-mutant cell lines are colored. **(C)** Volcano plot comparing the IC_{50} difference between *BRAF* p.V600E-mutant and *BRAF* p.V600E wild-type cell lines for the 34 inhibitors. Green nodes indicate inhibitors which are significantly more active in *BRAF* p.V600E-mutant compared to *BRAF* p.V600E wild-type cell lines, as determined by MANOVA. Red nodes indicate inhibitors which are significantly less active in *BRAF* p.V600E-mutant cell lines.

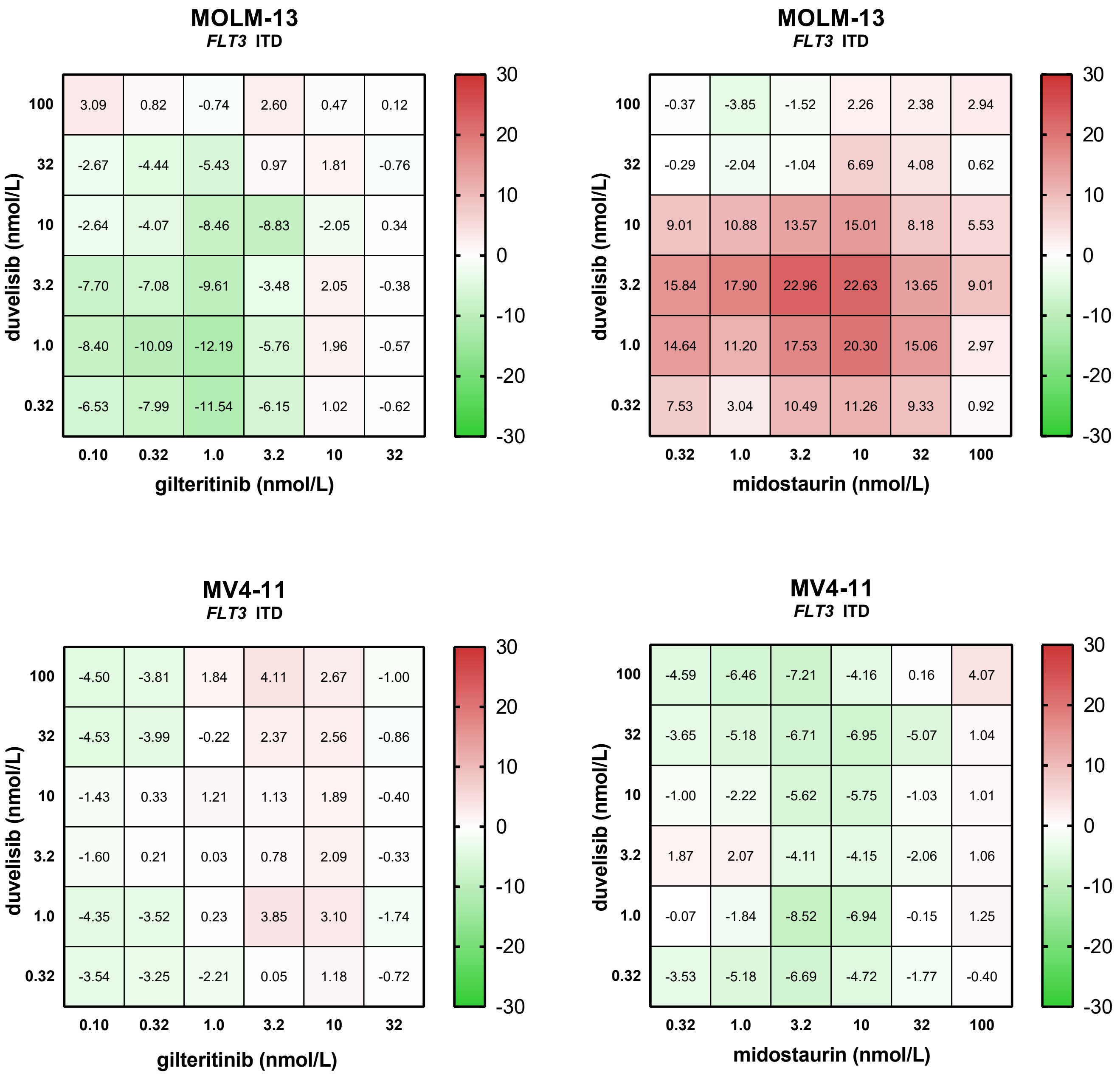
MOLM-13
FLT3 ITD
duvelisib (nmol/L)
100 3.09 0.82 -0.74 2.60 0.47 0.12
32 -2.67 -4.44 -5.43 0.97 1.81 -0.76
10 -2.64 -4.07 -8.46 -8.83 -2.05 0.34
3.2 -7.70 -7.08 -9.61 -3.48 2.05 -0.38
1.0 -8.40 -10.09 -12.19 -5.76 1.96 -0.57
0.32 -6.53 -7.99 -11.54 -6.15 1.02 -0.62
0.10 0.32 1.0 3.2 10 32
gilteritinib (nmol/L)
30 20 10 0 -10 -20 -30
MOLM-13
FLT3 ITD
duvelisib (nmol/L)
100 -0.37 -3.85 -1.52 2.26 2.38 2.94
32 -0.29 -2.04 -1.04 6.69 4.08 0.62
10 9.01 10.88 13.57 15.01 8.18 5.53
3.2 15.84 17.90 22.96 22.63 13.65 9.01
1.0 14.64 11.20 17.53 20.30 15.06 2.97
0.32 7.53 3.04 10.49 11.26 9.33 0.92
0.32 1.0 3.2 10 32 100
midostaurin (nmol/L)
30 20 10 0 -10 -20 -30
MV4-11
FLT3 ITD
duvelisib (nmol/L)
100 -4.50 -3.81 1.84 4.11 2.67 -1.00
32 -4.53 -3.99 -0.22 2.37 2.56 -0.86
10 -1.43 0.33 1.21 1.13 1.89 -0.40
3.2 -1.60 0.21 0.03 0.78 2.09 -0.33
1.0 -4.35 -3.52 0.23 3.85 3.10 -1.74
0.32 -3.54 -3.25 -2.21 0.05 1.18 -0.72
0.10 0.32 1.0 3.2 10 32
gilteritinib (nmol/L)
30 20 10 0 -10 -20 -30
MV4-11
FLT3 ITD
duvelisib (nmol/L)
100 -4.59 -6.46 -7.21 -4.16 0.16 4.07
32 -3.65 -5.18 -6.71 -6.95 -5.07 1.04
10 -1.00 -2.22 -5.62 -5.75 -1.03 1.01
3.2 1.87 2.07 -4.11 -4.15 -2.06 1.06
1.0 -0.07 -1.84 -8.52 -6.94 -0.15 1.25
0.32 -3.53 -5.18 -6.69 -4.72 -1.77 -0.40
0.32 1.0 3.2 10 32 100
midostaurin (nmol/L)
30 20 10 0 -10 -20 -30

Figure S2. Heatmaps of ZIP synergy scores for the 6x6 combination series of the FLT3 inhibitors gilteritinib and midostaurin combined with the PI3Kγ/δ inhibitor duvelisib in the *FLT3* ITD-mutant cell lines MOLM-13 (top) and MV4-11 (bottom). The ZIP score indicates the percentage of additional cell line response induced by the combination compared to the expected response based on the two single agents. A ZIP score > 10 was considered synergistic, from -10 to 10 was considered additive, while < -10 was considered antagonistic. The ZIP scores are based on four replicates.

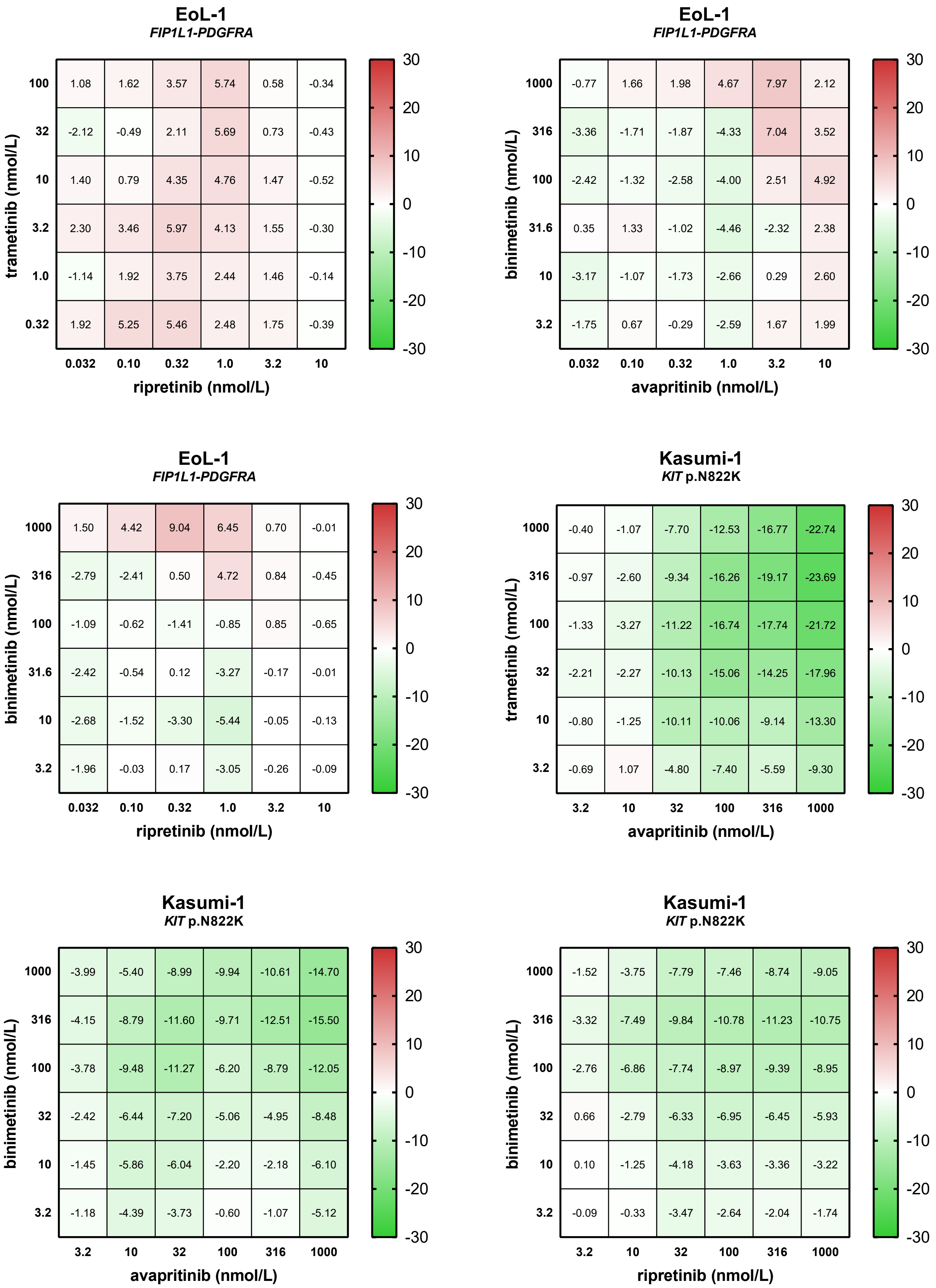
EoL-1
FIP1L1-PDGFRA
trametinib (nmol/L)
100: 1.08 1.62 3.57 5.74 0.58 -0.34
32: -2.12 -0.49 2.11 5.69 0.73 -0.43
10: 1.40 0.79 4.35 4.76 1.47 -0.52
3.2: 2.30 3.46 5.97 4.13 1.55 -0.30
1.0: -1.14 1.92 3.75 2.44 1.46 -0.14
0.32: 1.92 5.25 5.46 2.48 1.75 -0.39
0.032 0.10 0.32 1.0 3.2 10
ripretinib (nmol/L)
30 20 10 0 -10 -20 -30
EoL-1
FIP1L1-PDGFRA
binimetinib (nmol/L)
1000: -0.77 1.66 1.98 4.67 7.97 2.12
316: -3.36 -1.71 -1.87 -4.33 7.04 3.52
100: -2.42 -1.32 -2.58 -4.00 2.51 4.92
31.6: 0.35 1.33 -1.02 -4.46 -2.32 2.38
10: -3.17 -1.07 -1.73 -2.66 0.29 2.60
3.2: -1.75 0.67 -0.29 -2.59 1.67 1.99
0.032 0.10 0.32 1.0 3.2 10
avapritinib (nmol/L)
30 20 10 0 -10 -20 -30
EoL-1
FIP1L1-PDGFRA
binimetinib (nmol/L)
1000: 1.50 4.42 9.04 6.45 0.70 -0.01
316: -2.79 -2.41 0.50 4.72 0.84 -0.45
100: -1.09 -0.62 -1.41 -0.85 0.85 -0.65
31.6: -2.42 -0.54 0.12 -3.27 -0.17 -0.01
10: -2.68 -1.52 -3.30 -5.44 -0.05 -0.13
3.2: -1.96 -0.03 0.17 -3.05 -0.26 -0.09
0.032 0.10 0.32 1.0 3.2 10
ripretinib (nmol/L)
30 20 10 0 -10 -20 -30
Kasumi-1
KIT p.N822K
trametinib (nmol/L)
1000: -0.40 -1.07 -7.70 -12.53 -16.77 -22.74
316: -0.97 -2.60 -9.34 -16.26 -19.17 -23.69
100: -1.33 -3.27 -11.22 -16.74 -17.74 -21.72
32: -2.21 -2.27 -10.13 -15.06 -14.25 -17.96
10: -0.80 -1.25 -10.11 -10.06 -9.14 -13.30
3.2: -0.69 1.07 -4.80 -7.40 -5.59 -9.30
3.2 10 32 100 316 1000
avapritinib (nmol/L)
30 20 10 0 -10 -20 -30
Kasumi-1
KIT p.N822K
binimetinib (nmol/L)
1000: -3.99 -5.40 -8.99 -9.94 -10.61 -14.70
316: -4.15 -8.79 -11.60 -9.71 -12.51 -15.50
100: -3.78 -9.48 -11.27 -6.20 -8.79 -12.05
32: -2.42 -6.44 -7.20 -5.06 -4.95 -8.48
10: -1.45 -5.86 -6.04 -2.20 -2.18 -6.10
3.2: -1.18 -4.39 -3.73 -0.60 -1.07 -5.12
3.2 10 32 100 316 1000
avapritinib (nmol/L)
30 20 10 0 -10 -20 -30
Kasumi-1
KIT p.N822K
binimetinib (nmol/L)
1000: -1.52 -3.75 -7.79 -7.46 -8.74 -9.05
316: -3.32 -7.49 -9.84 -10.78 -11.23 -10.75
100: -2.76 -6.86 -7.74 -8.97 -9.39 -8.95
32: 0.66 -2.79 -6.33 -6.95 -6.45 -5.93
10: 0.10 -1.25 -4.18 -3.63 -3.36 -3.22
3.2: -0.09 -0.33 -3.47 -2.64 -2.04 -1.74
3.2 10 32 100 316 1000
ripretinib (nmol/L)
30 20 10 0 -10 -20 -30

Figure S3. Heatmaps of ZIP synergy scores for the 6x6 combination series of the PDGFRα and KIT inhibitors avapritinib and ripretinib, combined with one of the MEK inhibitors trametinib or binimetinib in the cell lines EoL-1 (*FIP1L1-PDGFRA*) and Kasumi-1 (*KIT* p.N822K). The ZIP score indicates the percentage of additional cell line response induced by the combination compared to the expected response based on the two single agents. A ZIP score > 10 was considered synergistic, from -10 to 10 was considered additive, while < -10 was considered antagonistic. The ZIP scores are based on four replicates.
